# Redescription of the forgotten New Caledonian weevil genus *Callistomorphus* Perroud, 1865 (Coleoptera, Curculionidae, Eugnomini) with descriptions of eight new species

**DOI:** 10.3897/zookeys.821.29019

**Published:** 2019-01-31

**Authors:** Miłosz Adam Mazur

**Affiliations:** 1 Institute of Biology, University of Opole, Oleska 22, 45–052 Opole, Poland University of Opole Opole Poland

**Keywords:** Beetles, biodiversity, endemic species, New Caledonia, new taxa, taxonomy, weevils

## Abstract

*Callistomorphus* is one of the “forgotten” genera of the tribe Eugnomini inhabiting rain forest in New Caledonia. In this paper, the genus *Callistomorphus* and the type species *C.farinosus* are redescribed. Eight new species, *Callistomorphusfundatus***sp. n.**, *C.gibbus***sp. n.**, *C.malleus***sp. n.**, *C.minimus***sp. n.**, *C.rutai***sp. n.**, *C.szoltysi***sp. n.**, *C.torosus***sp. n.** and *C.turbidus***sp. n.**, are described, originating from the main island of New Caledonia. Illustrations and SEM photographs of the external morphology and the male and female terminalia are provided, as well as dorsal habitus colour photographs of the adults, a key to the species, a distribution map, and a discussion of the systematic position of *Callistomorphus* within the tribe.

## Introduction

For many years, only three genera of Eugnomini from New Caledonia were known: *Pactola* Pascoe, 1876, with two species (a third was synonymised by Mazur (2014)), originally placed in the genus *Macropoda* Montrouzier, 1861 (see Mazur 2014); *Acanthopterus* Faust, 1889, with seven species (recently transferred to the tribe Aterpini by Kuschel (2014)); and a monotypic genus *Callistomorphus* Perroud, 1865.

Lacordaire (1863) established two groups for genera currently placed in Eugnomini (Alonso-Zarazaga and Lyal 1999): Eugnomides (as species group No. V within the Erirrhinides) for the genera *Eugnomus* Schoenherr, 1847, *Hypselus* Schoenherr, 1843 (now in the tribe Erirhinini), *Rhopalomerus* Blanchard, 1849, *Stephanorhynchus* White,1846, *Meriphus* Erichson, 1842, *Ophthalmoborus* Schoenherr, 1843 (now a synonym of *Anthobius* Schoenherr, 1833 (Derelomini)), *Phyllotrox* Schoenherr, 1843 (now in the tribe Derelomini) and *Brachonyx* Schoenherr, 1825 (now in the tribe Anthonomini); and Scoloptérides (as a tribe) for one genus – *Scolopterus* White, 1846.

*Callistomorphus* was described three years after the fundamental work of Lacordaire was published (Perroud and Montrouzier 1865). Although both Perroud and Montrouzier are stated as being authors, the descriptions of all Curculionidae, including the new genus and species *C.farinosus*, were written only by Perroud. This is evidenced by Perroud’s comment in this paper, and the fact that the text was written in the first person singular (see comment on page 248 in Alonso-Zarazaga and Lyal 1999). The new genus and species were classified as Eugnomides (sensu Lacordaire), closely related to *Stephanorhynchus*.

Since that time, the systematic position of the genus *Callistomorphus* has not been discussed in detail. In Junk’s “Coleopterorum Catalogus” (Pars, 140) (Klima 1934), the genus was clearly placed in Eugnomini (at that time, a subdivision of the Erirrhininae); subsequently, the genus was practically forgotten and ignored by further researchers.

It is probable that Voss, who studied the Eugnomini in the 1930s and gave them subfamily status, did not examine any *Callistomorphus* specimens. He mentioned the genus only once (1937), vaguely indicating its similarity to the genus *Macropoda* (now a synonym of *Pactola*, see above). Subsequently, he pointed out the necessity for a closer examination of the genus and its affiliation to Stephanorhynchina, which was the species group established by him one year earlier (Voss 1936) for two genera: *Stephanorhynchus* and *Hoplocneme* White, 1846 (including neither *Callistomorphus* nor *Macropoda*). It is likely that Voss examined only species from New Zealand, and that his comparisons were based on the original description of *Callistomorphus* with no specimens at hand.

A more detailed study of the Eugnominae was carried out by Marshall (1937). He introduced a more comprehensive diagnosis to the subfamily established by Voss, and he drew up a key for the New Zealand genera. He also considered the Australian genus *Meriphus* Erichson, 1842 and some of its relatives, and established the new subfamily Meriphinae for them. Additionally, he added comments on the other genera of Eugnomini, e.g. Chilean *Rhopalomerus* (currently also known from New Zealand); but again, *Callistomorphus*, as well as *Macropoda*, were not mentioned in this work.

Another comprehensive taxonomic paper on the Eugnominae was that of Cawthra (1966). He examined most of the known genera, redefined the subfamily, and summarised the available information about the biology and distribution of the studied species. This author also drew up a key for genera not considered by Marshall (those known from outside New Zealand). Once again, *Callistomorphus* was not included in the study and was mentioned only as: "*a genus occurring in New Caledonia, not included in Marshall’s key and, according to the original description of Perroud, very similar to* Stephanorrhynchus *White, from which it differs by the fact that its rostrum is three times, not twice, as long as the head*".

May (1993) analysed in detail the morphology of the larvae from six genera of Eugnominae and revised the status of the subfamily to a tribe with two subtribes – Eugnomina Lacordaire, 1863 and Meriphina Marshall, 1937. This state of affairs was confirmed by Alonso-Zarazaga and Lyal (1999).

In this paper, a redescription of the genus *Callistomorphus* is presented, as well as descriptions of eight new species from New Caledonia, along with a key to all the species within the genus and comments about the taxonomic position of the genus within the tribe.

## Materials and methods

This study is based on 26 specimens. Holotypes are deposited in the Muséum National d’Histoire Naturelle, Paris (MNHN). Paratypes are deposited in the Museum of Natural History, University of Wrocław, Poland (MNHW) and in the Natural History Department of Upper Silesian Museum, Bytom, Poland (USMB).

Measurements were made using a calibrated stereomicroscopic grid eyepiece (C-W10xB/22) in a Nikon SMZ-800 stereomicroscope. Genitalia preparations were made according to the standard method of macerating the separated abdomen for 5–10 min in a warm KOH solution. After dissection, if necessary, terminal structures were stained with a solution of Chlorazol Black E in glycerine for 5–10 min under visual control. Habitus photographs were taken using a Canon Power Shot A640 camera connected with the stereomicroscope and processed using the Helicon Focus v. 4.50 and PhotoFiltre v. 6.1 software programmes. All drawings were made by using the Corel Draw package. Scanning electron micrographs were taken using a Hitachi S-3400N.

The nomenclature of the male terminalia and abbreviations of particular measurements (partly modified) follows Wanat (2001):

**al** abdomen length (measured through the middle of ventrites);

**apw** pronotum width at anterior margin;

**arw** width of rostrum apex;

**aw** abdomen maximum width;

**bew** width of elytral base (measured through the middle of humeral calli);

**bpw** pronotum width at the base;

**el** elytra length (measured in top view in a position when the base and apex of elytra are at the same level);

**eyl** eye length (measured in top view, when the head is positioned horizontally);

**frw** minimum frons width;

**hl** head length (measured in top view, from anterior edge of pronotum to fore margin of eyes);

**hw** head width (measured across the middle of the eyes);

**lb** length of body exclusive of rostrum;

**lvl** last ventrite length (measured through the middle);

**lvw** last ventrite maximum width;

**mpw** minimum pronotal width;

**pl** pronotum length (measured through the middle);

**rl** rostrum length, measured in dorsal view, when base and apex of rostrum are at the same level;

**scl** antennal scape length.

All dimensions are given in millimetres.

The distribution maps (Fig. 145) use a base map obtained from the Demis World Maps Service, open source (http://www2.demis.nl/worldmap/mapper.asp).

## Taxonomy

### Genus Callistomorphus

Taxon classificationAnimaliaColeopteraCurculionidae

Perroud, 1865

Callistomorphus Perroud, 1865: Perroud and Montrouzier 1865 [1864-misprint]: 169 (description); Gemminger and Harold 1871: 2449 (catalogue); Marschall 1873: 178 (catalogue); Scudder 1882: 49 (catalogue of generic names in zoology); Klima 1934: 80 (catalogue); Alonso-Zarazaga and Lyal 1999: 79 (catalogue). Type species: Callistomorphusfarinosus Perroud, 1865 (by monotypy).

#### Diagnosis.

Distinguished from other genera of Eugnomini by the following combination of characters: rostrum elongate, longer than pronotum alone, but shorter than head and pronotum taken together; in dorsal view with distinct, polished longitudinal carina. Mandibles elongate, distinctly protruding beyond apical margin of rostrum, not exodont, overlapping. Head behind eyes distinctly constricted. Pronotum strongly narrowed before apical part with pair of various developed tubercles near middle of length. Elytra strongly scabrous with numerous, small tubercles and pair of large, elongate tubercles near middle of length (next as “middle tubercles”). Legs elongate, all femora strongly broadened, with distinct, enlarged tooth that is usually larger than half of maximum femoral width; all tibiae distinctly sinuate, without mucro in male; tarsal claws free at base, glabrous, only regularly extended basally.

#### Redescription.

Body length (lb) – 7.20–14.70 mm.

*Body colour and vestiture* (Figs 13–21). Entire body covered with strictly adjoining, small, elongate scales. Colour variable but general patterns appear stable in some species (e.g. *C.farinosus* Perroud). Middle of elytra usually with paler spot between the 7^th^ and 11^th^ elytral intervals and usually extending from one-third to two-thirds of elytral length, though sometimes shorter.

**Figures 1–12.**
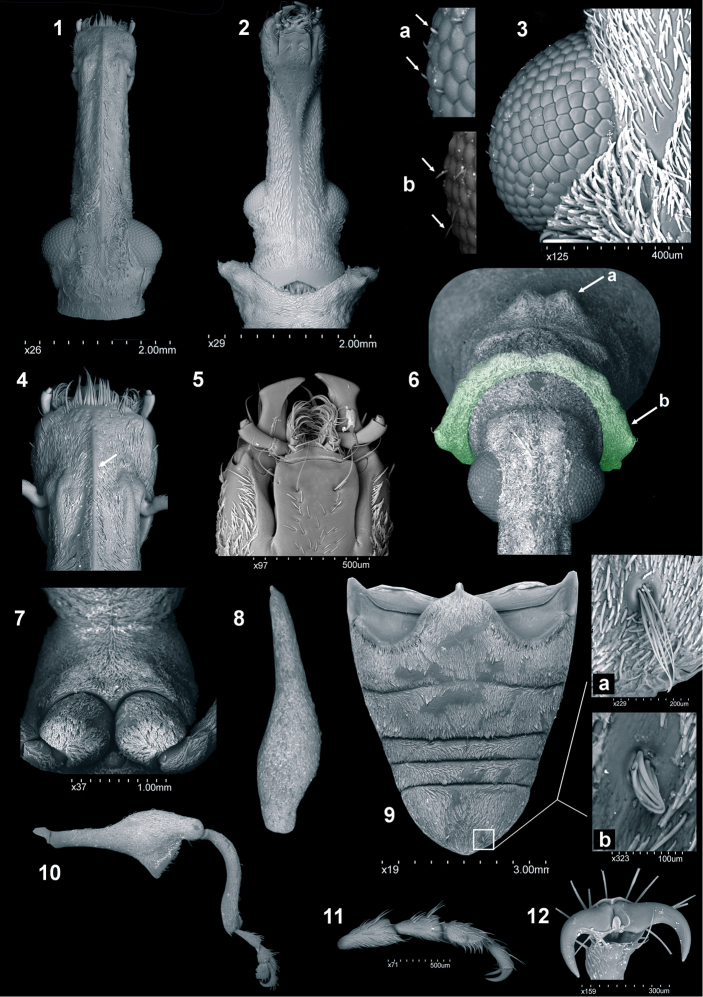
General morphology of *Callistomorphus*: **1***C.farinosus* Perr., head and rostrum, dorsal view **2***C.malleus* sp. n., head and rostrum ventral view **3***C.fundatus* sp. n., ventral view of eye with magnification of setae between ommatidia (**a***C.fundatus* sp. n., **b***C.gibbus* sp. n.) **4***C.farinosus* Perr., apical part of rostrum with antennal insertion, dorsal view **5***C.turbidus* sp. n., mouth parts **6***C.gibbus* sp. n., head and pronotum, frontodorsal view; a medial tubercles on pronotum, b thickened front “wall” of pronotum (greened) **7***C.rutai* sp. n., fore coxae **8***C.gibbus* sp. n., hind femur, dorsal view **9***C.fundatus* sp. n., abdomen **a***C.torosus* sp. n. **b***C.fundatus* sp. n., apical setae on last ventrite **10***C.malleus* sp. n., hind leg, lateral view **11***C.turbidus* sp. n., hind tarsus **12***C.rutai* sp. n., tarsal claws.

*Rostrum* (Figs 1, 2, 4, 5, 58–75). Elongate, almost as long as head and pronotum taken together; medially in most species with distinct longitudinal carina (Fig. 4) reaching almost to hind margin of eyes, slightly to distinctly curved in lateral view (Figs 58–66), maximum width at apical portion between antennal insertion and apex, slightly narrowing from antennal insertion to base. *Scrobes* partially visible in dorsal view (Fig. 4); in lateral view visible to about two-thirds of length; in ventral view dilated, not connected, evanescent before fore margin of eyes (Fig. 2). *Antennae* elongate; scape reaches beyond front margin of eye, funicle 7-segmented, club elongate (Figs 67–75). *Mouthparts* (Fig. 5) with elongate, flexible maxillae, reaching distinctly beyond front margin of rostrum; maxillary palpi 3-segmented, second maxillary palpomere distinctly elongate, third segment very short. Labial palps three-segmented, third palpomere very small, slightly protruding from second palpomere. Mentum short and wide, 3–4 × wider than long. Submentum slightly longer than wide. Mandibles strongly elongate, distinctly protruding beyond edge of rostrum; overlapping, with one apical incisor; outer edge gently rounded inwardly; inner edge smooth, without teeth.

*Head* (Figs 1, 2, 3a, b, 6, 58–66, 76–84). Subquadrate to transverse, distinctly narrowed behind eyes. Vertex usually with a pair of small tubercles, each furnished with bundle of scales. Eyes slightly to strongly convex, only in *C.minimus* sp. n. protruding above margin of head in lateral view; setae between some ommatidia short (shorter than diameter – e.g. in *C.fundatus* sp. n.) or elongate (longer than diameter – e.g. in *C.gibbus* sp. n.). Gular suture in most species visible.

*Pronotum* (Figs 6a, b, 40–57). With characteristic shape: broadest at base, distinctly narrowed to more or less two-thirds of length and strongly expanded apically (except *C.minimus* sp. n.). Apical margin in most species strongly scabrous with a few (8–10) tubercles on dorsal and lateral edge; anterior surface of pronotum forming distinct flat wall (visible in anterior view) (Fig. 6b). In lateral view, a pair of conspicuously protruding or flattened tubercles are present near midpoint (Figs 6a, 49–57). Anterior area, in lateral view, with transverse groove and sparse, shallow punctures (Figs 49–57).

*Elytra* (Figs 22–39). Longer than wide (el/bew 1.47–1.68) with eleven intervals. Widest at base, through the middle of well-developed humeral calli; lateral margins subparallel to ca. fourth-fifths of length before strongly narrowing to apex. Third interval, near middle of length, with distinct tubercle (except *C.minimus* sp. n.); height of the tubercle subequal to width of two or three intervals, the length more or less from one-third to one-quarter length of elytra. Intervals convex, in some species intervals 3, 5, 7, 9 more convex with numerous, irregular, small tubercles (flattened or acuminate). Seventh interval narrowed on short distance behind humeral angles, apically with more or less distinct tubercle (next as – posterior calli), protruding beyond outline of elytra in dorsal view (except *C.minimus* sp. n.); 9^th^ interval behind humeral angles weakly protruding, clearly visible in dorsal view on this section.

*Legs* (Figs 7, 8, 10, 11, 12). Fore coxae contiguous (Fig. 7). Femora elongate at base and strongly broadened medially with enlarged tooth (Figs 8, 10). Tooth on fore femora with margins and apex obtuse, middle and hind with apex acute and sharp outer edge. All tibiae elongate, slender, distinctly sinuate (Fig. 10). Tarsi elongate, as long as 0.5 × length of tibiae; first tarsomere slightly longer than second, second and third of similar length. Claws untoothed, broadened basally (Figs 11, 12).

*Abdomen* (Figs 9a, b, 85–93, 101–107, 127–131). Subquadrate to longer than wide, al/aw 0.93–1.20. First suture fused medially, sometimes obsolete; sutures between ventrites 2–5 strongly depressed. Last ventrite short (except *C.fundatus* sp. n.) apically with pair of bundled, erect setae (Fig. 9a, b); in most species with shallow depressions laterally and apically between pair of erect setal tufts. Margin of last ventrite usually with distinct, sharp edge (Fig. 91). Pygidium concealed by elytra; in male generally subquadrate (Figs 101–107); in female wider than its length (Figs 127–131).

*Male terminalia* (Figs 94–100, 108–121). Penis (Figs 94–100) well sclerotised, distinctly curved in lateral view; basal piece (apodemal bridge) from weakly (e.g. *C.farinosus*) to fully sclerotised (e.g. *C.turbidus* sp. n.).

Apodemes shorter than penis body; basally narrow, than distinctly extended, laterally flattened.

*Tegmen* (Figs 108–114). With elongate apodeme, parameroid lobes divided in different ways (detailed in description of species).

*Spiculum gastrale* (Figs 115–121). With elongate apodeme and divided base. Hemisternites strongly sclerotised, in most species directly connected with base of spiculum.

*Female terminalia* (Figs 122–126, 132–144). Abdominal tergite VIII (Figs 122–126) usually subtriangular with maximum width at base (exception – *C.farinosus*). Lateral edges with conspicuous, strongly elongate setae. Spermatheca (Figs 132–134) strongly curved. Abdominal sternite VIII (Figs 135–139) with elongate apodeme and well-developed apical lobe with species-specific patterns of sclerotisation. Lateral edges of apical lobe with short, erect setae. Ovipositor (Figs 140–144) with elongate gonocoxite; styli elongate, apically with one or two bundles of elongate setae.

*Sexual dimorphism*. *Callistomorphus* is a genus with a very indistinct sexual dimorphism. Specimens within particular species vary in size and proportion of the body and values of these parameters overlap each other (Tab. 1). Both sexes have a similar last ventrite; length of rostrum and proportion of elytra are not diagnostic.

*Distribution*. The genus is endemic in New Caledonia, known only from the main island, Grande Terre. Localities where particular species were collected are shown in Fig. 145.

*Biology*. The detailed biology of species is unknown. Although other members of Eugnomini have been reared from dead wood, subcortical tissues, live stems, galls, and the leaves or fruits of many species of plants from different families (e.g. May 1987, Mazur et al. 2016), specimens of *Callistomorphus* were collected by beating or by sifting from the litter. Many species are suspected to have nocturnal activity, often being collected using light traps or by beating vegetation at night (see the data from the labels). According to the label data and the personal comments of Marek Wanat, members of the genus are most commonly found on plant leaves in humid and rain forest growing on limestone and/or ultramafic rocks (Bonvallot et al. 2013), some of them only at altitudes exceeding 500 metres above sea level.

*Remarks*. Members of the genus are variable in terms of their size, body proportions and colour. However, they are separated from the other genera of the tribe by the set of characters presented in the above diagnosis. Many of the species are also the biggest members of the tribe. Despite their large size and characteristic body form, members of this genus are not common in museum collections or in the field. For example, during the French fieldwork conducted in the 1980s and 1990s, where fogging and standard collecting methods were used (pers. com. Hélène Perrin), no single specimen of *Callistomorphus* was found (see the label data of the specimens deposited in MNHN); fewer than 30 specimens of the genus were recently collected during three Polish expeditions (2006, 2008 and 2010) where a wide range of collecting methods (beating vegetation at night and day, sifting, sweep netting, light traps) were used. Most of these specimens represent new species which are described in this paper.

**Table 1.** Indices for species of *Callistomorphus* Perr., where: m – male, f – female.

	* C. farinosus *	* C. fundatus *	* C. gibbus *	* C. malleus *	* C. minimus *	* C. rutai *	* C. szoltysi *	* C. torosus *	* C. turbidus *
hw/hl	m: 1.07	m: 1.07	m: 1.00–1.20	m: 1.00–1.20	f: 1.17	m: 1.08	f: 1.08	m: 0.92	m: 0.92
	f: 1.00–1.10			f: 1.00–1.33				f: 1.00	
eyl/hl	m: 0.50–0.53	m: 0.43	m: 0.45–0.55	m: 0.50–0.60	f: 0.56	m: 0.50	f: 0.50	m: 0.38	m: 0.42
	f: 0.47–0.53			f: 0.45–0.55				f: 0.46	
rl/pl	m: 1.09–1.12	m: 1.04	m: 1.00–1.12	m: 1.17–1.21	f: 1.21	m: 1.20	f: 1.20	m: 1.12	m: 1.11
	f: 1.03–1.11			f: 1.12–1.24				f: 1.07	
rl/arw	m: 4.00–4.22	m: 3.38	m: 3.00–3.17	m: 3.80–4.30	f: 3.40	m: 4.00	f: 3.35	m: 3.50	m: 2.86
	f: 3.67–4.00			f: 4.00–4.50				f: 3.33	
scl/rl	m: 0.74–0.78	m: 0.85	m: 0.82–0.90	m: 0.75–0.80	f: 0.76	m: 0.80	f: 0.80	m: 0.82	m: 0.75
	f: 0.82–0.83			f: 0.70–0.85				f: 0.83	
bpw/pl	m: 0.97–1.03	m: 1.04	m: 1.06–1.15	m: 1.00–1.05	f: 1.21	m: 1.08	f: 1.20	m: 1.04	m: 1.17
	f: 0.97–1.00			f: 0.95–1.10				f: 1.11	
bpw/apw	m: 1.46–1.52	m: 1.35	m: 1.26–1.47	m: 1.25–1.35	f: 1.70	m: 1.35	f: 1.43	m: 1.30	m: 1.24
	f: 1.55–1.64			f: 1.28–1.38				f: 1.35	
mpw/apw	m: 0.71	m: 0.65	m: 0.63–0.71	m: 0.66–0.68	f: 0.90	m: 0.65	f: 0.67	m: 0.65	m: 0.59
	f: 0.70–0.73			f: 0.61–0.69				f: 0.61	
mpw/bpw	m: 0.47–0.49	m: 0.48	m: 0.48–0.50	m: 0.48–0.53	f: 0.53	m: 0.45	f: 0.47	m: 0.50	m: 0.48
	f: 0.45–0.49			f: 0.48–0.50				f: 0.45	
el/bew	m: 1.52	m: 1.65	m: 1.48–1.55	m: 1.56–1.66	f: 1.68	m: 1.55	f: 1.55	m: 1.51	m: 1.47
	f: 1.48–1.58			f: 1.50–1.68				f: 1.55	
al/aw	m: 1.04-1.06	m: 1.18	m: 0.94-1.20	m: 1.08-1.17	f: 1.15	m: 1.05	f: 0.98	m: 1.00	m: 1.06
	f: 1.02-1.06			f: 1.06-1.20				f: 0.93	
lvw/lvl	m: 2.20-2.50	m: 1.75	m: 2.14-2.37	m: 2.25-2.57	f: 2.60	m: 2.75	f: 2.27	m: 2.56	m: 2.13
	f: 2.33-2.55			f: 2.13-2.38				f: 2.78	

### Callistomorphus farinosus

Taxon classificationAnimaliaColeopteraCurculionidae

Perroud, 1865

Figs 1 , 4 , 13 , 22 , 31 , 40 , 49 , 58 , 67 , 76 , 85 , 94 , 101 , 108 , 115 , 122 , 127 , 132 , 135 , 140

Callistomorphus farinosus Perroud, 1865: 170, pl. 1, fig. 7

#### Diagnosis.

The largest member of the genus. Last three antennomeres of funicle wider than long. Apical margin of pronotum widely concave medially in dorsal view. Elytra with characteristic whitish spot in the area from medial tubercles to 7^th^ intervals, not reaching apical part. Penis body distinctly, regularly narrowed from base to apex in lateral view. Parameroid lobes of tegmen slightly divided apically. Female abdominal tergite VIII with maximum width near middle.

#### Redescription.

Body length (lb) – 12.80–14.70 mm.

**Figures 13–21.**
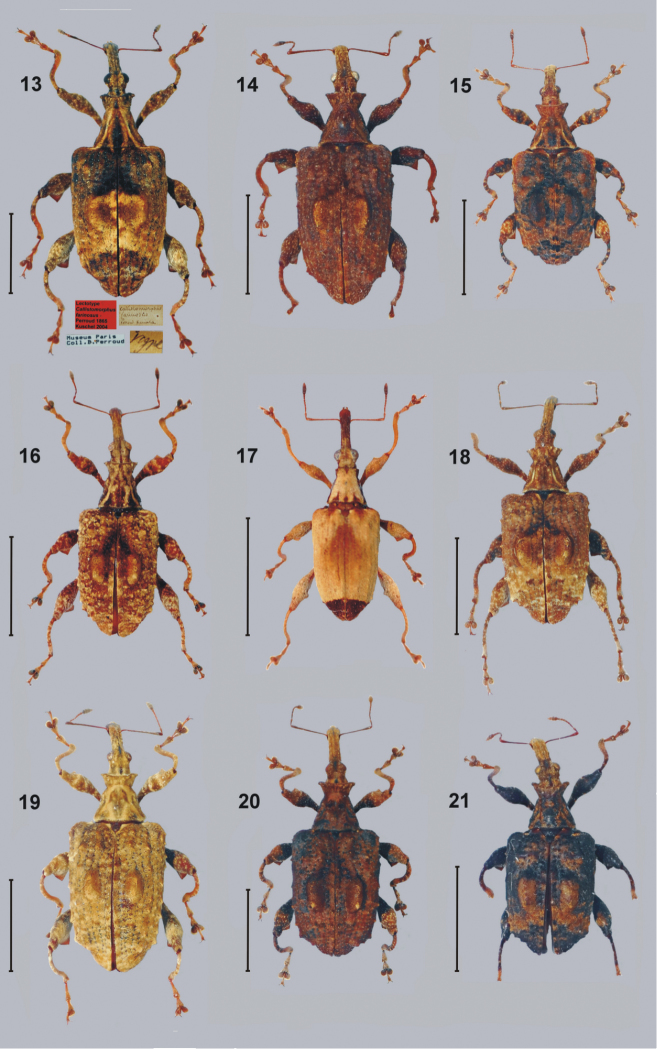
Dorsal habitus colour photographs of New Caledonian species from the genus *Callistomorphus*: **13***C.farinosus* Perr., lectotype, female with original labels **14***C.fundatus* sp. n., male, holotype **15***C.gibbus* sp. n., holotype, male **16***C.malleus* sp. n., paratype, female **17***C.minimus* sp. n., holotype, female **18***C.rutai* sp. n., holotype, male **19***C.szoltysi* sp. n., holotype, female **20***C.torosus* sp. n., paratype, female **21***C.turbidus* sp. n., holotype, male. Scale bar = 5 mm.

*Body colour and vestiture* (Fig. 13). As stated in diagnosis, whitish spot on elytra is clearly visible on generally darker ground coloration. Some specimens with T- or X-shaped blackish spot on elytra, extending from base to medial tubercles. Pronotum with distinct, light, oblique lines. All studied additional specimens also with distinctly lighter hind legs than lectotype. Darker area at apical part of elytra sometimes with transverse stripe of paler scales.

*Head* (Figs 1, 4, 58, 67, 76). Subquadrate to slightly longer than wide (hw/hl ♂: 1.07; ♀: 1.00–1.10). Frons as wide as 2 × width of eye in dorsal view. Eyes approximately as long as half-length of head (eyl/hl ♂: 0.50–0.53; ♀: 0.47–0.53), not protruding above margin of head in lateral view. Rostrum slightly longer than pronotum (rl/pl ♂: 1.09–1.12; ♀: 1.03–1.11), 3.50–4.10 × as long as width of rostrum apex; distinct, polished carina on entire length; regularly curved in lateral view. Scape shorter than rostrum (scl/rl = ♂: 0.74–0.78; ♀: 0.82–0.83). First funicle segment ca 1.8 × as long as 2^nd^, and almost as long as 2^nd^ and 3^rd^ taken together; 3^rd^ shorter than 2^nd^; from 4^th^ to 7^th^ funicle segments as long as wide or little longer. Club almost as long as 2 × maximum width; as long as last four funicle segments combined.

*Pronotum* (Figs 40, 49). Subquadrate (bpw/pl ♂: 0.97–1.03; ♀: 0.97–1.00). Base slightly sinuate; approximately 1.50–1.60 × as wide as apical margin (bpw/apw ♂: 1.46–1.52; ♀: 1.55–1.64); apically with single tubercle at angles, medial portion widely incised. Width of medial constriction in relation to apical and basal margin: mpw/apw ♂: 0.71; ♀: 0.70–0.73, mpw/bpw ♂: 0.47–0.49; ♀: 0.45–0.49. Medial tubercle on pronotal disc wide and glabrous, sometimes weakly developed. Apical area beyond medial tubercles lies distinctly lower than base (clearly visible in lateral view); in lateral view apical margin medially extended forward with rounded apex.

**Figures 22–30.**
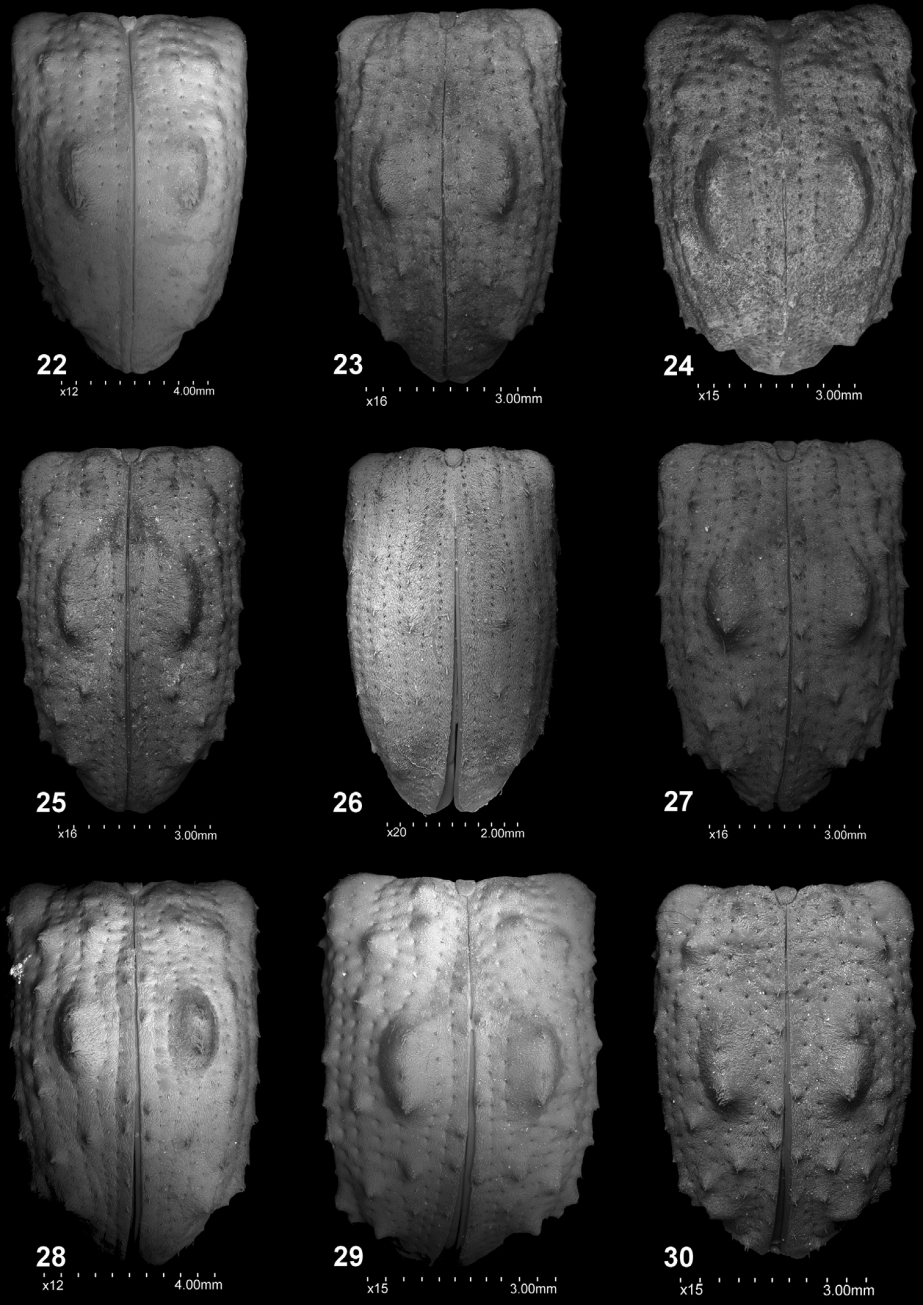
Elytra, dorsal view: **22***C.farinosus* Perr. **23***C.fundatus* sp. n. **24***C.gibbus* sp. n. **25***C.malleus* sp. n. **26***C.minimus* sp. n. **27***C.rutai* sp. n. **28***C.szoltysi* sp. n. **29***C.torosus* sp. n. **30***C.turbidus* sp. n.

**Figures 31–39.**
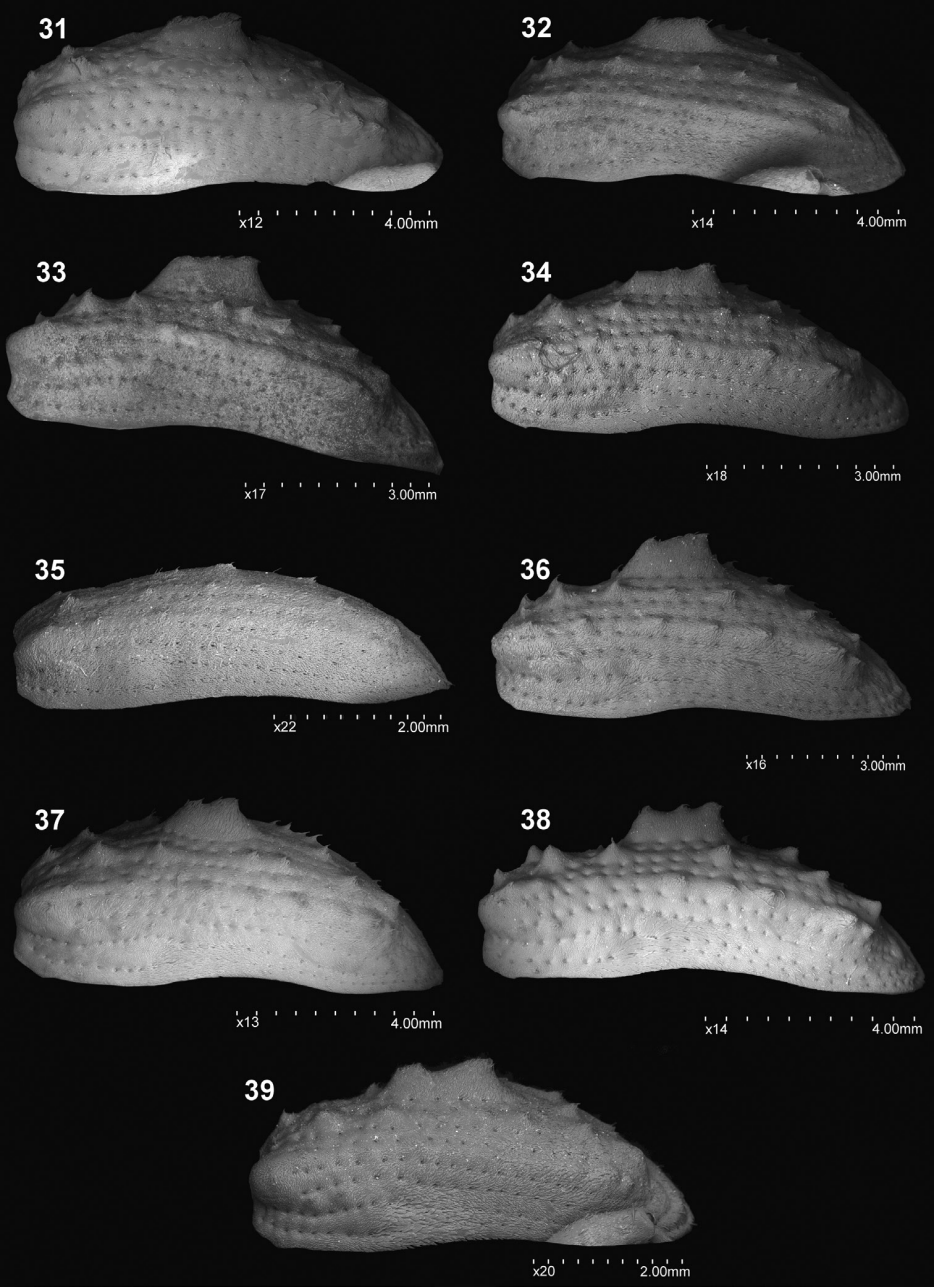
Elytra, lateral view: **31***C.farinosus* Perr. **32***C.fundatus* sp. n. **33***C.gibbus* sp. n. **34***C.malleus* sp. n. **35***C.minimus* sp. n. **36***C.rutai* sp. n. **37***C.szoltysi* sp. n. **38***C.torosus* sp. n. **39***C.turbidus* sp. n.

*Elytra* (Figs 22, 31). Approximately 1.5 × as long as its width (el/bew ♂: 1.52; ♀: 1.48–1.58). Distinctly narrowed from humeral calli to apical part. Medial tubercles distinct, narrow, wider than width of 3^rd^ interval; hind angle with higher elevation than fore, furnished with bundle of dense setae. Striae with single line of suboval punctures. Variable tubercles on odd intervals obtuse. Scutellum subtriangular.

*Abdomen* (Figs 85, 101). Abdomen slightly longer than wide (al/aw ♂: 1.04–1.06; ♀: 1.02–1.06). Pygidium as in Fig. 101, distinctly narrowed before apical part. Last ventrite short, more than 2 × wider than long (lvw/lvl ♂: 2.20–2.50; ♀: 2.33–2.55).

*Male terminalia* (Figs 94, 108, 115). Penis body little longer than apodemes; in dorsal view slightly narrowed to rounded apex; in lateral view distinctly curved at base; regularly narrowing apicad; apodemal bridge weakly sclerotised. Endophallus everted without visible sclerites. Parameroid lobes of tegmen divided apically, with similar length as apodeme. Spiculum gastrale with hooked basal piece. Hemisternites on spiculum gastrale irregular, small, strongly sclerotised.

*Female terminalia* (Figs 122, 127, 132, 135, 140). Apodeme of sternite VIII with separate basal part; apical lobe with characteristic shape of sclerotisation occupying medial area. Abdominal tergite VIII slightly broadened from base to more or less middle of length, remainder of tergite VIII distinctly narrowed to rounded apex; lateral margin with elongate setae. Spermatheca as in Fig. 132. Pygidium with maximum width at base, distinctly narrowed to rounded apex. Ovipositor with relatively small gonocoxite; apical setae short; vagina stout.

*Measurements*. ♂: al 4.80–5.00, apw 2.10–2.40, arw 0.90, aw 4.60–4.70, bew 5.80–6.00, bpw 3.20–3.50, el 8.80–9.10, eyl 0.70–0.80, frw 0.70–0.80, hl 1.40–1.50, hw 1.50–1.60, lb 13.60–13.70, lvl 0.90–1.00, lvw 2.20–2.30, mpw 1.50–1.70, pl 3.30–3.40, rl 3.60–3.80, scl 2.80.

♀: al 4.70–5.40, apw, 2.00–2.20, arw 0.90–1.00, aw 4.40–5.20, bew 5.30–6.40, bpw 3.10–3.60, el 8.40–9.50, eyl 0.70–0.80, frw 0.60–0.70, hl 1.50, hw 1.50–1.65, lb 12.80–14.70, lvl 0.90–1.00, lvw 2.10–2.40, mpw 1.40–1.60, pl 3.20–3.60, rl 3.30–4.00, scl 2.70–3.30.

#### Type material.

Lectotype, 1♀ (here designated, see Remarks) – “*Callistomorphusfarinosus*, Perroud Kanala” – handwritten; “type” – handwritten; “♀” – printed; small red circle; Lectotype *Callistomorphusfarinosus* Perroud, 1865; Museum Paris, coll. B. Perroud (MNHN).

#### Additional material.

1♀ – New Caledonia (S); 22°01.9'S, 166°28.0'E, Dzumac Mts 900 m (Mt Ouin road junction), 28.12.2006 night collecting, leg. M. Wanat & R. Dobosz (MNHW).

1♂ – New Caledonia (N), 20°57'19.1"S, 165°17'27.”E, Pic d’Amoa (Povilla), 18.11.2010 450 m, end 0.5 km of road, leg. M. Wanat, R. Ruta (MNHW).

1♂ – New Caledonia (S), 22°02'13.6"S, 166°29'44.5"E, Mt. Dzumac (base), 1.5–3 km E of Ouin rd jct., 6.12.2010, 800 m, rainforest, leg. M. Wanat, R. Ruta (MNHW).

1♀ – New Caledonia (N); 20°57'23.7"S, 165°17'27.7"E, Pic d’Amoa (Povila), 450 m, end 0.5 km of road, 22.11.2008, leg. M. Wanat (MNHW).

#### Remarks.

Kuschel selected a female specimen from Perroud’s original series as a syntype in 2004 but this action was never published. To ensure stability in nomenclature and to clarify identity of this species I herein designate the same female specimen as the lectotype. I take this action under the article 74.1 of the Code (ICZN).

### Callistomorphus fundatus sp. n.

Taxon classificationAnimaliaColeopteraCurculionidae

http://zoobank.org/ECEF260B-10D9-48EF-A421-8E5FAFC567F6

Figs 3 , 9 , 14 , 23 , 32 , 41 , 50 , 59 , 68 , 77 , 86 , 95 , 102 , 109 , 116

#### Diagnosis.

This species can be distinguished from other members of the genus by the following set of characters: elytra elongate, 1.6 × as long as wide across humeral calli; colour of body generally brown; pronotum with apical margin almost straight, corrugated due to numerous, small tubercles, basal margin concave; last ventrite less than 2 × wider than long with shallow apical depression.

#### Description.

Body length (lb) – 11.40 mm.

*Body colour and vestiture* (Fig. 14). Generally brown with yellow shade in some areas. Striae and intervals each with single line of elongate yellowish setae. Legs with sparse setae of the same colour as elytra.

*Head* (Figs 3, 59, 68, 77). Subquadrate (♂: hw/hl = 1.07). Frons 2 × as wide as eye in dorsal view. Eyes shorter than half length of head (♂: eyl/hl = 0.43), not protruding above margin of head in lateral view. Rostrum slightly longer than pronotum (♂: rl/pl = 1.04), 3.38 × as long as maximum width at apex (rl/arw); with thin, polished carina on entire length; regularly curved in lateral view. Scape shorter than rostrum (♂: scl/rl = 0.85). First funicle segment approximately 1.4 × as long as 2^nd^; 3^rd^ approximately 0.65 as long as 2^nd^; antennomeres from 4^th^ to 7^th^ successively little shorter than previous one, longer than wide. Club elongate, approximately 3.8 × as long as wide, as long as last four funicle segment combined.

*Pronotum* (Figs 41, 50). Slightly longer than wide (♂: bpw/pl = 1.04). Basal margin distinctly, widely concave; 1.35 × as wide as apical margin (♂: bpw/apw); apically straight with numerous, small tubercles; in lateral view apical margin straight anteriorly, then converging towards base. Medial tubercle on pronotal disc well developed, divided into two, strongly protruding appendices. Width of medial constriction in relation to apical and basal margin: ♂: mpw/apw = 0.65, ♂: mpw/bpw = 0.48.

*Elytra* (Figs 23, 32). Elongate (♂: el/bew = 1.65). Slightly narrowed from humeral calli to apical part. Medial tubercles distinct, higher than width of its base on 3^rd^ interval. Striae with single line of suboval punctures. Odd intervals with distinct, tooth-shape, tubercles with elongate, slightly hooked, single scale on the top. Tubercles on 7^th^ intervals conspicuous, protruding from outline of elytra in dorsal view. Scutellum distinctly elongate, ca 1.5 × as long as wide.

*Abdomen* (Figs 9b, 86, 102). Abdomen of male 1.18 × as long as wide (al/aw). Male pygidium as in Fig. 102. Last ventrite elongate, subtriangular, lvw/lvl = 1.75.

*Male terminalia* (Figs 95, 109, 116). Penis body distinctly longer than apodemes; almost subparallel from base to apical part, apically slightly narrowed to rounded apex; basal part partly sclerotised, except medial area; in lateral view distinctly, regularly curved, apically slightly upturned. Internal sac longitudinally crinkled, without any apparent structure or sclerites. Parameroid lobes of tegmen slightly shorter than apodeme, divided almost to base; the dorsal part of the tegminal ring with membrane and sharp, protruding process. Tegminal apodeme apically extended. Spiculum gastrale robust, slingshot-shape, apically strongly sclerotised; in lateral view apodeme apically curved; hemisternites fused with base of spiculum.

*Female* – unknown

*Measurements*. ♂: al 4.50, apw, 2.00, arw 0.80, aw 3.80, bew 4.80, bpw 2.70, el 7.90, eyl 0.60, frw 0.70, hl 1.40, hw 1.50, lb 11.40, lvl 1.20, lvw 2.10, mpw 1.30, pl 2.60, rl 2.70, scl 2.30.

#### Type material.

Holotype, ♂ (here designated) – New Caledonia (N); 20°57.2'S, 165°17.5'E; Pic d’Amoa 360 m; 14.01.2007 forest at light; leg. M. Wanat & R. Dobosz (MNHN).

#### Etymology.

This epithet is derived from the Latin word “*funda*” (slingshot) and refers to the shape of spiculum gastrale. A variable adjective.

#### Remarks.

Only one specimen of this new species has been found within the studied collections. A set of characteristic features, including the almost uniform brown colour, elongate elytra and last ventrite, as well as terminal structures, indicates that this is a new species.

### Callistomorphus gibbus sp. n.

Taxon classificationAnimaliaColeopteraCurculionidae

http://zoobank.org/56D2BC10-7248-448F-8E13-8DBDF447CE08

Figs 6 , 15 , 24 , 33 , 42 , 51 , 60 , 69 , 78 , 87 , 96 , 103 , 110 , 117

#### Diagnosis.

This species can be distinguished from other members of the genus by the following suite of characters: apical margin of pronotum concave in dorsal view, base sinuate, protruding towards elongate scutellum. Rostrum distinctly bent in middle of length.

#### Description.

Body length (lb) – 7.90–9.60 mm.

*Body colour and vestiture* (Fig. 15). Generally dark brown with various, irregular spots of different shades of brown. Legs in some specimens with mottled coloration, from whitish to variable shades of orange and almost black. Tibiae generally paler, more or less orange. Pronotum sometimes with darker spots near base, medially and with pair of narrow, oblique stripes of white-yellow scales from hind angles to middle of length.

*Head* (Figs 6, 60, 69, 78). Subquadrate to wider than long (♂: hw/hl = 1.00–1.20). Frons wider than double width of eye. Eyes approximately as long as half of length (♂: eyl/hl = 0.45–0.55), not protruding above margin of head in lateral view, regularly rounded. Rostrum as long as pronotum or little shorter (♂: rl/pl = 1.00–1.12), from 3.00 to 3.17 × as long as maximum width (rl/arw); with polished, sharp longitudinal carina on entire length; in lateral view distinctly curved medially. Scape shorter than rostrum (♂: scl/rl = 0.82–0.90). First funicle segment slightly longer than 2^nd^, 3^rd^ almost as half-length of 2^nd^, antennomeres from 4^th^ to 7^th^ with similar length, little longer than wide. Club slightly as long as last four funicle segment combined, ca 1.8 × as long as wide.

*Pronotum* (Figs 6, 42, 51). Wider than long (♂: bpw/pl = 1.06–1.15); base 1.26–1.47 × as wide as apical margin (bpw/apw); apical margin distinctly, widely concave with small tubercles; apical angles with rounded, distinct tubercle; in lateral view apical margin protruding towards head. Medial tubercle on pronotal disc well developed, divided into two, strongly protruding appendices. Width of medial constriction in relation to apical and basal margin in male: mpw/apw = 0.63–0.71, mpw/bpw = 0.48–0.50.

*Elytra* (Figs 24, 33). Approximately 1.5 × as long as its width (♂: el/bew = 1.48–1.55); slightly narrowed from humeral calli to apical part. Medial tubercles distinct, elongate; elytral disc with numerous, small acuminate tubercles; tubercles on 7^th^ intervals easily visible, protruding from outline of elytra in dorsal view. Striae with suboval, deep punctures; surface of elytral disc distinctly rugose. Scutellum triangular, as long as wide at base.

*Abdomen* (Figs 87, 103). In male from 0.94 to 1.20 × as long as wide (al/aw). Male pygidium as in Fig. 87. Last ventrite 2.14–2.37 × wider than long (lvw/lvl).

*Male terminalia* (Figs 96, 110, 117). Penis body slightly longer than apodemes; from base to apical part almost subparallel, apically slightly narrowed to rounded apex; basal part sclerotised, except medial part; distinctly, regularly curved in lateral view, apices thin and distinctly upturned. Internal sac without any structure or sclerites. Parameroid lobes of tegmen thin, distinctly shorter than apodeme, divided almost to base. Tegminal apodeme apically extended. Spiculum gastrale robust, similar to *C.fundatus* sp. nov; hemisternites fused with base of spiculum.

*Female* – unknown.

*Measurements*. ♂: al 3.20–3.60, apw, 1.40–1.90, arw 0.60–0.70, aw 2.90–3.50, bew 3.40–4.20, bpw 1.80–2.50, el 5.30–6.50, eyl 0.50–0.60, frw 0.50–0.60, hl 0.90–1.10, hw 1.00–1.20, lb 7.90–9.60, lvl 0.70–0.85, lvw 1.50–2.00, mpw 0.90–1.20, pl 1.70–2.20, rl 1.80–2.20, scl 1.60–1.80.

#### Type material.

Holotype, ♂ (here designated) – New Caledonia (N); 21°08'56.0"S, 165°19'20.9"E; Aoupinié (refuge), 400 m, at light; 25.11.2006; leg. M. Wanat (MNHN).

Paratypes: 1♂ – New Caledonia (N); 21°08.9'S, 165°19.4'E; Aoupinié (refuge), 18.01.2007, 420 m, at light, leg. M. Wanat & R. Dobosz (MNHW).

1♂ – New Caledonia (N), 20°57.2'S, 165°17.5'E, Pic d’Amoa, 360 m, 14.01.2007, forest at light, leg. M. Wanat & R. Dobosz (MNHW).

1♂ – New Caledonia (S), 22°01'54.5"S, 166°28'02.6"E, Mt. Ouin Rd, 900 m, 0–0.5 km N of Dzumac jct, 5.12.2010, night coll., leg. M. Wanat & R. Dobosz (MNHW).

#### Etymology.

This epithet is the Latin noun “*gibbus*” (protuberance, hump) and refers to a pair of large tubercles on elytra. A noun in apposition.

#### Remarks.

The shape of pronotum together with the lateral profile of the rostrum are characteristic for this new species. The terminalia are quite similar to *C.fundatus* sp. n. but differ in the shape of the apex of the penis (in dorsal view more rounded, in lateral view more upwardly directed in *C.gibbus* sp. n.). One small specimen was quite similar to *C.turbidus* sp. n. in bodily proportions, but distinctly different in the shape of the pronotum, rostrum length and form of terminal structures.

### Callistomorphus malleus sp. n.

Taxon classificationAnimaliaColeopteraCurculionidae

http://zoobank.org/9722BE21-8800-4C58-BCED-E28EEE21E3FE

Figs 2 , 10 , 16 , 25 , 34 , 43 , 52 , 61 , 70 , 79 , 88 , 97 , 104 , 111 , 118 , 123 , 128 , 133 , 136 , 141

#### Diagnosis.

This species can be distinguished from other members of the genus by the following suite of characters: rostrum gently curved, regularly narrowed to apex in lateral view. Middle tubercles on elytra flattened, lower than maximal width at base. Apex of penis in lateral view expanded into small tubercles. Apical lobes of female abdominal sternite VIII with characteristic shape (Fig. 136).

**Figures 40–48.**
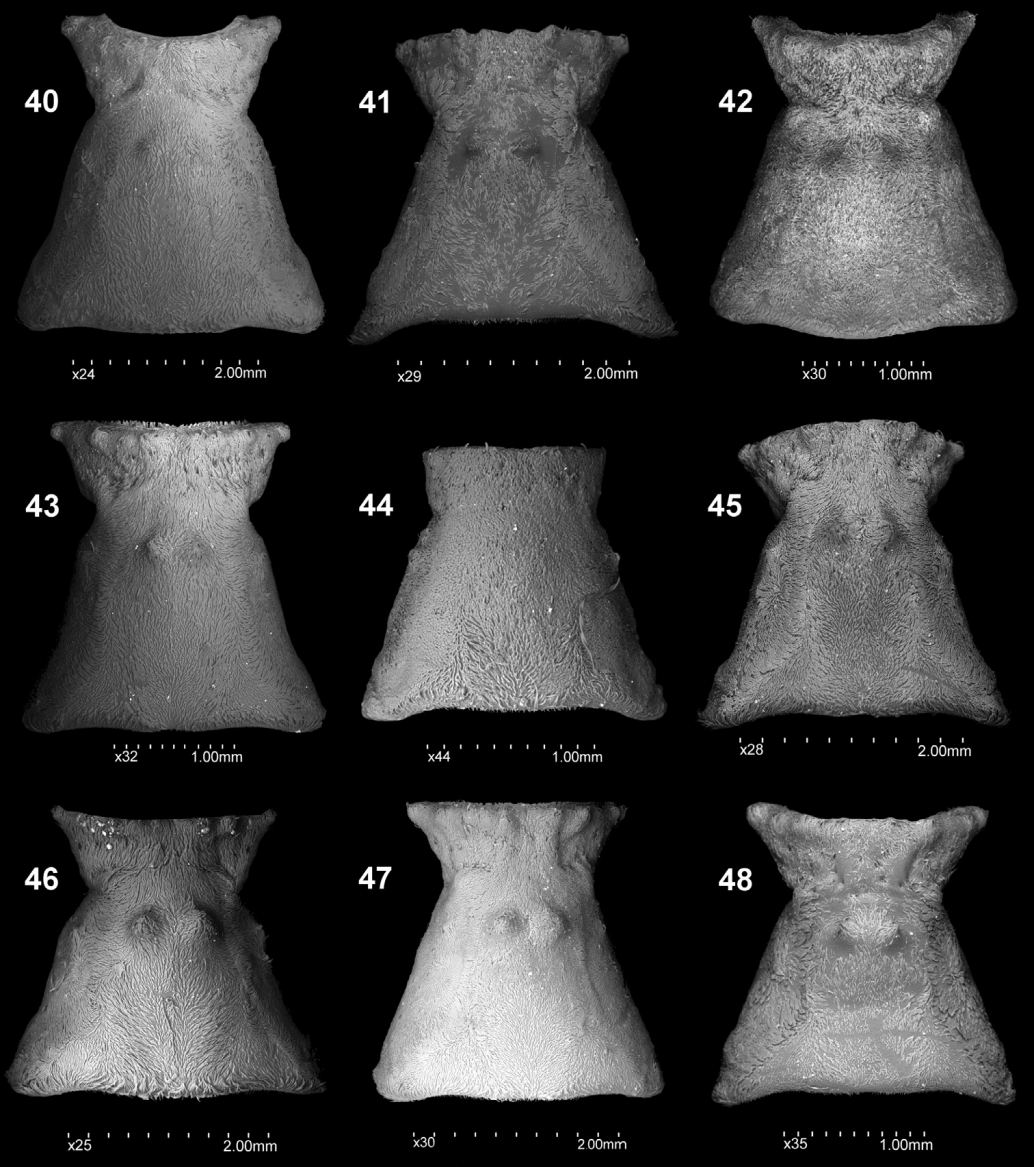
Pronotum, dorsal view: **40***C.farinosus* Perr. **41***C.fundatus* sp. n. **42***C.gibbus* sp. n. **43***C.malleus* sp. n. **44***C.minimus* sp. n.; **45***C.rutai* sp. n. **46** – *C.szoltysi* sp. n. **47***C.torosus* sp. n. **48***C.turbidus* sp. n.

#### Description.

Body length (lb) – 8.60–10.50 mm.

*Body colour and vestiture* (Fig. 16). Colour variable, from dark brown to yellowish. Some specimens with whitish coating on apical and lateral parts of elytra. Similar, pale coating, in some specimens, also covered hind femora. Pronotum with longitudinal yellowish stripes.

*Head* (Figs 61, 70, 79). From subquadrate to elongate (hw/hl ♂: 1.00–1.20; ♀: 1.00–1.33), depending on protrusion the head from pronotum. Frons wider than double width of eye. Eyes approximately half as long as head or little longer (eyl/hl ♂: 0.50–0.60; ♀: 0.45–0.55), not protruding above margin of head in lateral view; regularly convex, widest near middle of length. Rostrum longer than pronotum (rl/pl ♂: 1.17–1.21; ♀: 1.12–1.24); in male 3.80–4.30 × as long as maximum width, in female 4.00–4.50 × as long as wide at apex (rl/arw); longitudinal carina indistinct, from base to antennal insertion covered by scales, apically bare, flattened, sometimes evanescent and visible only as polished, narrowed area. Scape shorter than rostrum (scl/rl ♂: 0.75–0.80; ♀: 0.70–0.85). First funicle segment ca 1.3 × as long as 2^nd^; 3^rd^ almost as half-length of 2^nd^, antennomeres from 4^th^ to 7^th^ with similar length, slightly longer than wide. Club as long as last four funicle segment combined, approximately 2.10 × as long as wide.

*Pronotum* (Figs 43, 52). Subquadrate (bpw/pl ♂: 1.00–1.05; ♀: 0.95–1.10). Base in male 1.25–1.35 ×, in female 1.28–1.38 ×, as wide as apical margin (bpw/apw); apical margin straight or slightly concave; in lateral view apical margin straight anteriorly, then converging towards base. Medial tubercle on pronotal disc well developed but weakly protruding. Width of medial constriction in relation to apical and basal margin: mpw/apw ♂: 0.66–0.68; ♀: 0.61–0.69, mpw/bpw ♂: 0.48–0.53; ♀: 0.48–0.50.

*Elytra* (Figs 25, 34). Elongate (el/bew ♂: 1.56–1.66; ♀: 1.50–1.68); lateral margins subparallel basaly to apical convergence. Medial tubercle distinct but flattened, lower than width on base; smaller, numerous tubercles on elytral disc acuminate; tubercles on 7^th^ intervals clearly visible, protruding from outline of elytra in dorsal view. Striae with small, suboval, shallow punctures; surface of elytral disc slightly glabrous. Scutellum slightly longer than wide.

*Abdomen* (Figs 88, 104, 128). From slightly (in male) to distinctly (in female) longer than wide (al/aw ♂: 1.08–1.17; ♀: 1.06–1.20 × as long as wide. Male pygidium as in Fig. 104, female as in Fig. 129. Last ventrite longer than wide (lvw/lvl ♂: 2.25–2.57; ♀: 2.13–2.38).

*Male terminalia* (Figs 97, 111, 118). Penis body slightly longer than apodemes; from base to apical part almost subparallel, apically slightly narrowed to rounded apex; basal part sclerotised, except medial area; in lateral view distinctly, regularly curved, apically expanded into small tubercles. Internal sac without any structure or sclerites. Parameroid lobes of tegmen thin, distinctly shorter than apodeme, divided almost to base and surrounded by thin membrane. Tegminal apodeme apically extended. Spiculum gastrale robust, apodeme laterally flattened, distinctly bent distally; hemisternites fused with base of spiculum.

*Female terminalia* (Figs 123, 128, 133, 136, 141). Apical lobe of abdominal sternite VIII with characteristic, T-shape sclerotisation. Abdominal tergite VIII with elongate, straight, dense setae near apex; sides with strongly elongate setae; apex with small, pointed tubercles. Spermatheca elongate, strongly bent. Gonocoxite placed diagonally to partly sclerotised vagina, strongly elongate, apically with bundle of erect setae.

*Measurements*. ♂: al 3.50–4.00, apw, 1.60–1.80, arw 0.60–0.70, aw 3.00–3.7, bew 3.50–4.30, bpw 2.00–2.40, el 5.80–6.90, eyl 0.60, frw 0.40–0.50, hl 1.00–1.20, hw 1.15–1.20, lb 8.60–10.40, lvl 0.70–0.80, lvw 1.60–2.00, mpw 1.05–1.20, pl 1.90–2.40, rl 2.30–2.80, scl 1.80–2.20.

♀: al 3.80–4.10, apw, 1.60–1.80, arw 0.60–0.70, aw 3.30–3.80, bew 3.90–4.30, bpw 2.20–2.40, el 6.00–7.20, eyl 0.50–0.60, frw 0.50–0.60, hl 0.9–1.20, hw 1.10–1.25, lb 9.50–10.50, lvl 0.80, lvw 1.70–2.00, mpw 1.10–1.20, pl 2.10–2.50, rl 2.50–2.80, scl 1.90–2.10.

**Figures 49–57.**
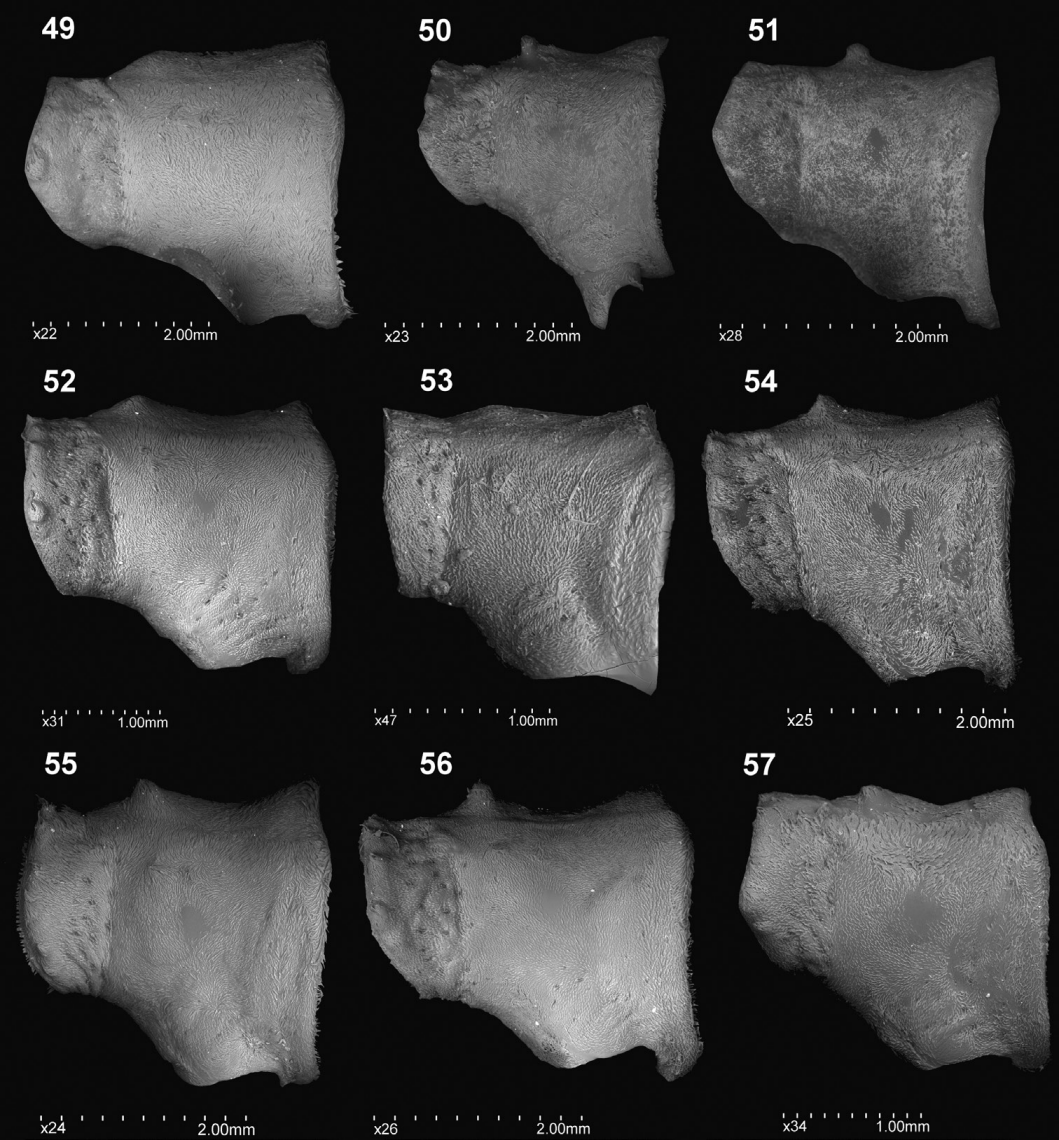
Pronotum, lateral view: **49***C.farinosus* Perr. **50***C.fundatus* sp. n. **51***C.gibbus* sp. n. **52***C.malleus* sp. n. **53***C.minimus* sp. n. **54***C.rutai* sp. n. **55***C.szoltysi* sp. n. **56***C.torosus* sp. n. **57***C.turbidus* sp. n.

#### Type material.

Holotype, ♂ (here designated) – New Caledonia (S); 22°04'08.9"S, 166°26'48.0"E; Dzumac road; S of Mts Couvélé rd jct; 870→670 m beating; 31.10.2008; leg. M. Wanat (MNHN).

Paratypes:

1♂ – New Caledonia (S); 22°05.9'S, 168°38.3'E; Riviére Bleue Parc; 23.12.2006, 190 m, refuge; sifting forest litter; leg. R. Dobosz (USMB).

1♂ – New Caledonia (S); 22°14.9'S, 166°49.7'E; Pic du Pin, base; 25.12.2006, 280 m, forest & plantation; leg. R. Dobosz & M. Wanat (USMB).

1♂ – New Caledonia (S); 22°10'19.2"S, 166°45'40.0"E; Bois du Sud, 220 m, at light; 25.10.2008; leg. M. Wanat (MNHW).

1♀ – New Caledonia (S); 22°10'22.4"S, 166°45'47.9"E; Bois du Sud, 220–250 m, beating along track entering forest reserve; 20.10.2008; leg. M. Wanat (MNHW).

1♀ – New Caledonia (S); 22°05.8'S, 166°40.2'E; Riviére Bleue: Gue de la; 22.12.2006, 140 m Pourina; night coll. (lamp & beating); leg. M. Wanat & R. Dobosz (MNHW).

1♀ – New Caledonia (S); 22°01.9'S, 166°28.0'E; Dzumac Mts, 900 m; Mt. Ouin, road junction; 28.12.2006, night collecting; leg. M. Wanat & R. Dobosz (MNHW).

2♀♀ – New Caledonia (S); 22°12'21.2"S, 166°40'46.9"E; Col des Deux Tétons, 30.10.2010; humid forest, 220–250 m; leg. M. Wanat & R. Ruta (MNHW).

1♀ – New Caledonia (S); 22°06.0'S, 166°39.3'E; Riviére Bleue, Pont Germain to kaori géant (left river side), 160–180 m; 22.01.2007; leg. M. Wanat (MNHW).

#### Etymology.

This epithet is derived from the Latin noun “*malleus*” (hammer) and refers to the shape of female sternite VIII. A noun in apposition.

#### Remarks.

The species is variable in size and colour but easily distinguished by the elongate elytra with relatively small medial tubercles and weakly curved rostrum. Also, penis and female sternite VIII are characteristic.

**Figures 58–66.**
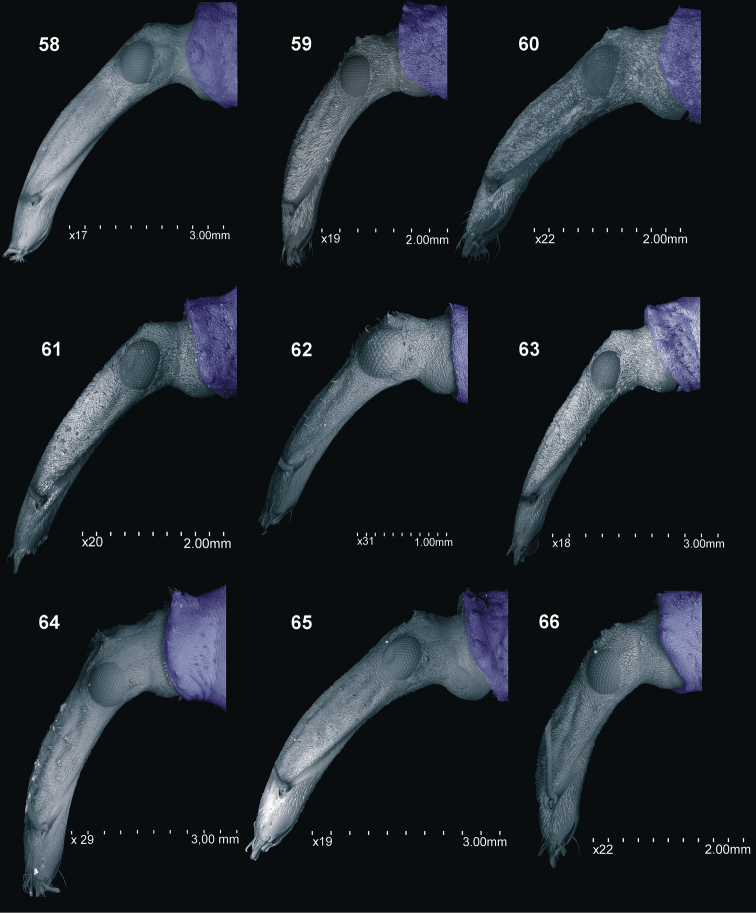
Head and rostrum, lateral view: **58***C.farinosus* Perr. **59***C.fundatus* sp. n. **60***C.gibbus* sp. n. **61***C.malleus* sp. n. **62***C.minimus* sp. n. **63***C.rutai* sp. n. **64***C.szoltysi* sp. n. **65** – *C.torosus* sp. n. **66** – *C.turbidus* sp. n.

### Callistomorphus minimus sp. n.

Taxon classificationAnimaliaColeopteraCurculionidae

http://zoobank.org/5D16E00A-66CC-441B-AD90-D0BF4399C7A6

Figs 17 , 26 , 35 , 44 , 53 , 62 , 71 , 80 , 89 , 124 , 129 , 137 , 142

#### Diagnosis.

The smallest member of the genus with several characteristic features. Eyes strongly convex, distinctly protruding above margin of head in lateral view. Pronotum distinctly narrowed from base to approximately three-quarters of length, apically sides only slightly expanded towards anterior margin; dorsal surface glabrous, medially only with small, obtuse tubercle. Elytra slender, elongate; without distinct medial tubercles, only with single, small tubercles on intervals. Apical part of elytra and sides of pronotum dark brown, in contrast to colour of the rest parts of body.

**Figures 67–75.**
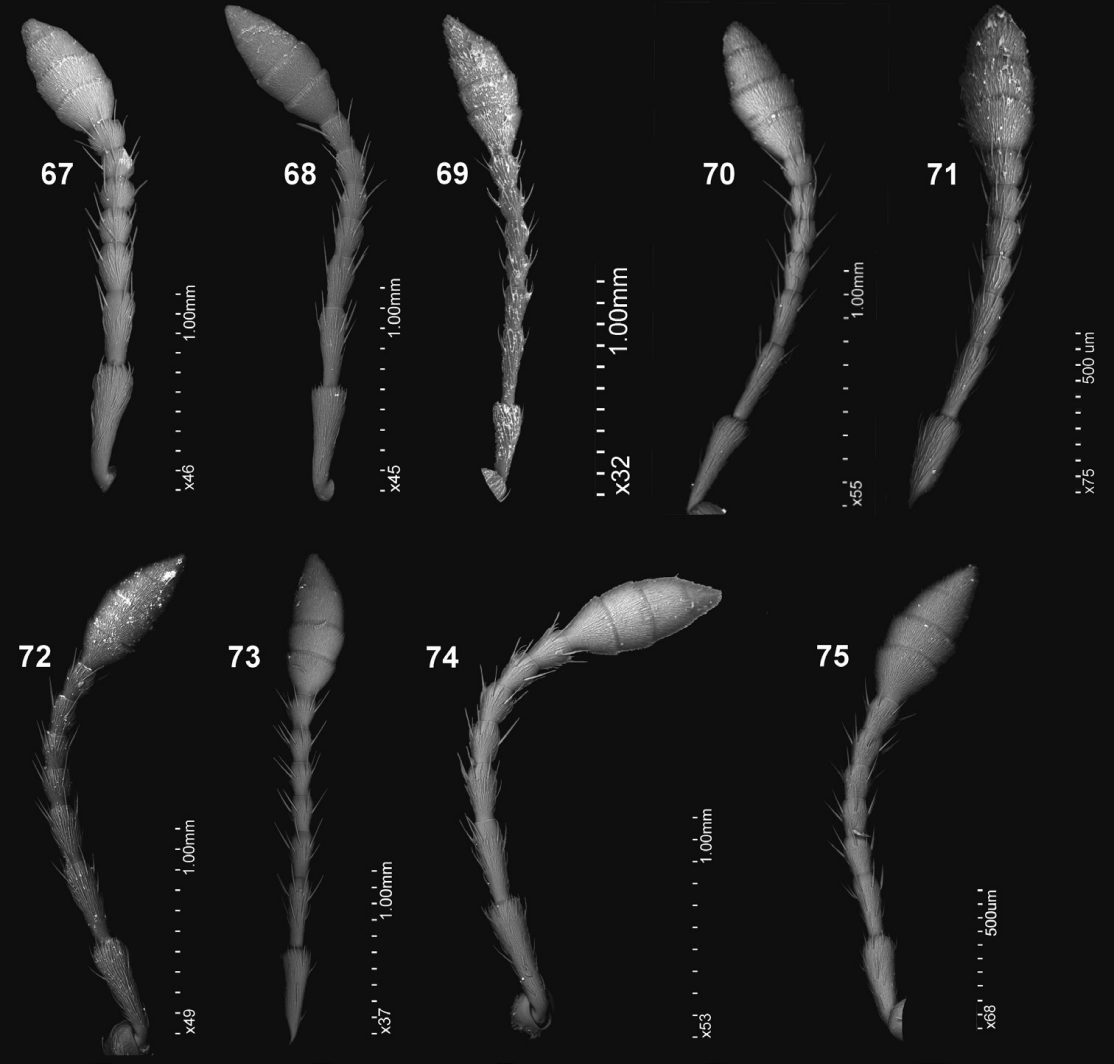
Antennae: **67***C.farinosus* Perr. **68***C.fundatus* sp. n. **69***C.gibbus* sp. n. **70***C.malleus* sp. n. **71***C.minimus* sp. n. **72***C.rutai* sp. n. **73***C.szoltysi* sp. n. **74***C.torosus* sp. n. **75***C.turbidus* sp. n.

#### Description.

Body length (lb) – 7.20 mm.

*Body colour and vestiture* (Fig. 17). Body covered almost entirely with yellowish scales. Rostrum brown. Antennae light brown. Lateral part of pronotum dark brown; base of pronotum in dorsal view with two, short, brownish stripes reaching to one-fifth of its length; between them small, brown spot. Elytra uniformly yellowish except: brownish apical angles ahead of humerus; base of intervals 3–5; indistinct, suboval darker spot from 1^st^ to 3^rd^ intervals before midlength. Apical part of elytra from four-fifth of length dark brown. Scutellum light brown. Mesepimeron, mesanepisternum and mesoventrite brown; metanepisternum together with metaventrite yellowish as most part of elytra. Legs uniformly yellowish.

*Head* (Figs 62, 71, 80). Slightly wider than long (hw/hl ♀: 1.17). Frons narrower than double width of eye; longitudinal carina between eyes distinct, surface between concave. Eyes strongly convex, circular, slightly longer than half length of head (eyl/hl ♀: 0.56), distinctly protruding above margin of head in lateral view. Rostrum longer than pronotum (rl/pl ♀: 1.20), 3.40 × as long as maximum width at apex (rl/arw); longitudinal carina indistinct, polished only from antennal insertion to apex. Scape shorter than rostrum (scl/rl ♀: 0.76). First funicle segment ca 1.2 × as long as 2^nd^ and 2 × as long as 3^rd^; antennomeres 4^th^ and 5^th^ slightly longer than wide; last two as long as wide. Club suboval, 2 × as long as wide, as long as last four funicle segment combined.

*Pronotum* (Figs 44, 53). Shorter than width at base (bpw/pl ♀: 1.21); 1.70 × as wide as apical margin (bpw/apw). Apical margin straight in dorsal view, not expanded, without tubercles; in lateral view anteriorly almost straight, then converging towards base; anterior transverse groove deeply concave. Medial tubercle not separate, only as slightly convex, single tubercle.

*Elytra* (Figs 26, 35). Elongate (el/bew ♀: 1.68). Regularly narrowed from base to apical part; posterior calli weakly developed, not protruding beyond outline of elytral in dorsal view. Surface of striae and intervals not visible due to very dense scales. Medial tubercles absent, on striae only single, small tubercles completely covered with scales. Scutellum slightly longer than wide, slightly protruding above margin of elytra in lateral view.

*Abdomen* (Figs 89, 129). Slightly longer than wide (al/aw 1.15). Second ventrite with sparse, erect, strongly elongate scales, clearly visible on the background of adjacent, shorter scales. Last ventrite 2.60 × wider than long (lv/lvl). Pygidium as in Fig. 129.

*Female terminalia* (Figs 124, 137, 142). Sternite VIII distinctly expanded apically with characteristic shape. Abdominal tergite VIII distinctly narrowed apically with rounded apex; sides with strongly elongate setae. Spermatheca lost in dissection. Ovipositor slender, almost straight; gonocoxite elongate; vagina well sclerotised.

*Male* – unknown

*Measurements*. ♀: al 3.00, apw 1.00, arw 0.50, aw 2.60, bew 2.85, bpw 1.70, el 4.80, eyl 0.50, frw 0.40, hl 0.80, hw 1.05, lb 7.40, lvl 0.50, lvw 1.30, mpw 0.90, pl 1.40, rl 1.70, scl 1.30.

**Figures 76–93.**
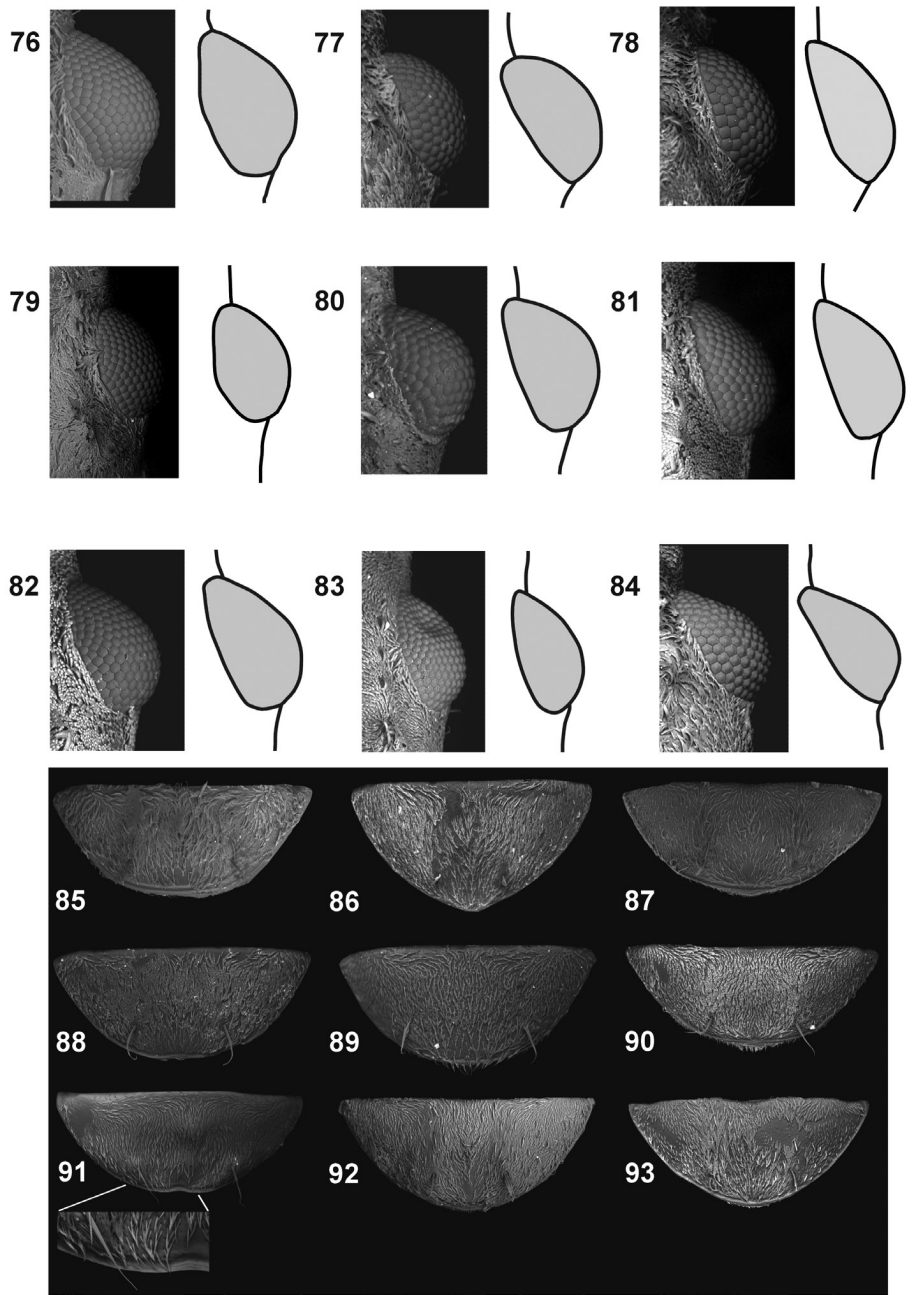
Outline of the eye, dorsal view: **76***C.farinosus* Perr., male **77***C.fundatus* sp. n., male **78***C.gibbus* sp. n. **79***C.malleus* sp. n. **80***C.minimus* sp. n. **81***C.rutai* sp. n. **82***C.szoltysi* sp. n. **83***C.torosus* sp. n. **84***C.turbidus* sp. n. Last ventrite: **85***C.farinosus* Perr., male **86***C.fundatus* sp. n., male **87***C.gibbus* sp. n., male **88***C.malleus* sp. n., male **89***C.minimus* sp. n., female **90** – *C.rutai* sp. n., male **91***C.szoltysi* sp. n., female **92***C.torosus* sp. n., male **93***C.turbidus* sp. n., male.

#### Type material.

Holotype, ♀ (here designated) – New Caledonia (S); 21°37'17.8"S, 165°52'38.6"E; Plateau de Dogny, 9.11.2010, 960 m; leg. R. Ruta (MNHN).

#### Etymology.

This epithet is the Latin adjective “*minimus*” (small, little), the new species is the smallest member of the genus.

#### Remarks.

*C.minimus* sp. n. is a very characteristic species. It is easy to distinguish from other members of the genus by small size, shape of pronotum (not extended apically), reduced tubercles on elytra and pronotum and contrasting coloration of the body.

### Callistomorphus rutai sp. n.

Taxon classificationAnimaliaColeopteraCurculionidae

http://zoobank.org/3CC352F4-668B-4C9C-8152-F554C3A8A00C

Figs 12 , 18 , 27 , 36 , 45 , 54 , 63 , 72 , 81 , 90 , 98 , 105 , 112 , 119

#### Diagnosis.

This species can be distinguished from other member of the genus by the following set of characters: medial tubercle on elytra very high; smaller tubercles numerous, very distinct and sharp; rostrum slender, slightly curved; penis narrowed before widely rounded apex; parameroid lobes of tegmen distinctly divided from midlength.

**Figures 94–107.**
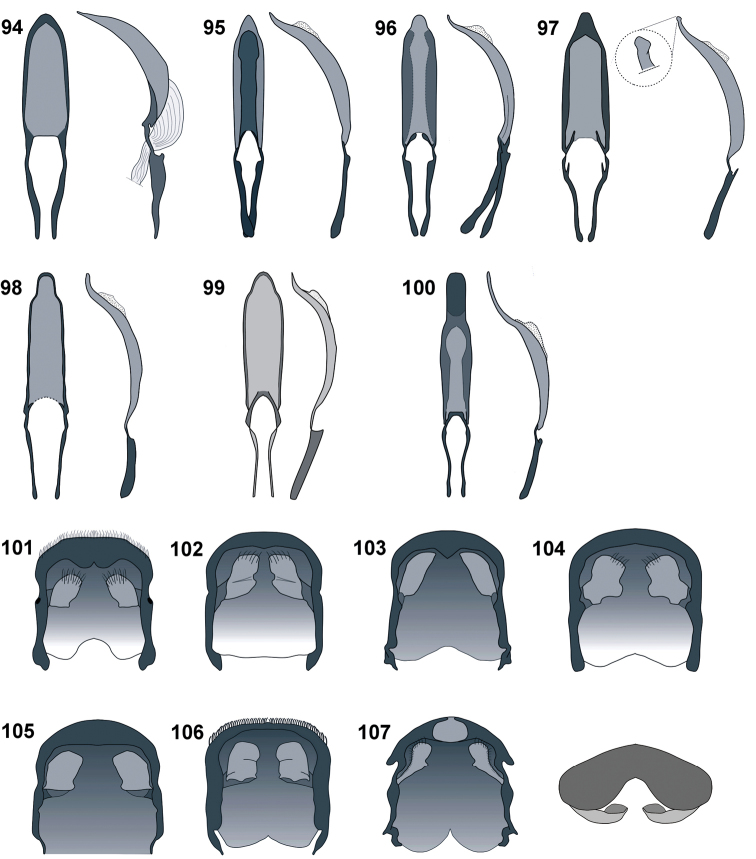
Penis: **94***C.farinosus* Perr. **95***C.fundatus* sp. n. **96***C.gibbus* sp. n. **97***C.malleus* sp. n. **98***C.rutai* sp. n. **99***C.torosus* sp. n. **100***C.turbidus* sp. n. Male pygidium, ventral view: **101***C.farinosus* Perr. **102***C.fundatus* sp. n. **103***C.gibbus* sp. n. **104***C.malleus* sp. n. **105***C.rutai* sp. n. **106***C.torosus* sp. n. **107***C.turbidus* sp. n – ventral and frontal view.

#### Description.

Body length (lb) – 10.80 mm.

*Body colour and vestiture* (Fig. 18). Colour variable, the body dappled with many small spots, from dark brown to yellowish, especially on distal part of elytra and hind legs. In front of medial tubercles on elytra darker spot from suture to base of tubercles. Pronotum with longitudinal yellowish stripes. Striae with distinct, single, short scales in each point of row.

*Head* (Figs 63, 72, 81). Slightly wider than long (♂: hw/hl = 1.08). Frons narrower than twice width of eye. Eyes convex, as long as half-length of head (eyl/hl ♂: 0.50), not protruding above margin of head in lateral view. Rostrum longer than pronotum (rl/pl ♂: 1.20), 4.00 × as long as maximum width at apex (rl/arw); longitudinal carina clearly visible only at apical part, posteriorly completely covered with scales. Scape shorter than rostrum (scl/rl ♂: 0.80). First funicle segment ca 1.30 × as long as 2^nd^; 3^rd^ 0.70 × as long as 2^nd^; antennomeres from 4^th^ to 7^th^ with similar length, elongate. Club 2.60 × as long as wide, as long as last four funicle segment combined.

*Pronotum* (Figs 45, 54). Slightly wider than long (bpw/pl ♂: 1.08). Base 1.35 × as wide as apical margin (bpw/apw); apical margin in dorsal view straight with numerous, distinct tubercles (on SEM photography (Fig. 45) apical margin of pronotum is seen as convex because the image was taken in different angle); in lateral view apical margin straight anteriorly, then converging towards base. Medial tubercle on pronotal disc distinct, strongly protruding, separate apically. Width of medial constriction in relation to apical and basal margin in male: mpw/apw = 0.65, mpw/bpw = 0.45.

*Elytra* (Figs 27, 36). Slightly more than 1.50 × as long as its width (el/bew ♂: 1.55). Subparallel from base to apical part; posterior calli distinct, strongly protruding beyond outline of elytral in dorsal view. Odd intervals with distinct, pointed tubercles that are easily visible in dorsal and lateral views, each furnished with single, hooked, elongate scale. Striae weakly impressed, formed by oval punctures, each with single, whitish scale inside. Medial tubercles distinct, strongly protruding, as high as almost half its length. Scutellum ca 1.20 × as long as wide.

*Abdomen* (Figs 90, 105). Slightly longer than wide (al/aw ♂: 1.05) in male. Last ventrite 2.75 × wider than long (lvw/lvl). Pygidium as in Fig. 105.

*Male terminalia* (Figs 98, 112, 119). Penis body distinctly longer than apodemes; almost subparallel from base to apical part; narrowed from fourth-fifth of length, apically widely rounded; basal part unsclerotised; distinctly, regularly curved in lateral view, apically strongly upturned, apex rounded. Internal sac without any structure or sclerites. Parameroid lobes of tegmen with extended common base, as long as apodeme, from half of length divided. Spiculum gastrale basally separate into two extended lobes; hemisternites indistinct.

*Female* – unknown

*Measurements*. ♂: al 4.30, apw 2.00, arw 0.75, aw 4.10, bew 4.80, bpw 2.70, el 7.40, eyl 0.60, frw 0.60, hl 1.20, hw 1.30, lb 10.80, lvl 0.80, lvw 2.20, mpw 1.30, pl 2.50, rl 3.00, scl 2.40.

#### Type material.

Holotype, ♂ (here designated) – New Caledonia (S); 22°11'S, 166°30'E; Koghi Mts.; humid forest, 500–550 m; 21.01.2004, leg. M. Wanat (MNHN).

#### Etymology.

This species is dedicated to my colleague Rafał Ruta, PhD (Wrocław, Poland), a great field researcher and specialist in Scirtidae (Coleoptera), who collected some specimens used in this paper, including the holotype of *C.minimus* sp. n.

#### Remarks.

In lateral view the head and rostrum are similar to those of *C.malleus* sp. n. (rostrum elongate, slightly curved). However, *C.rutai* sp. n. has more prominent medial tubercles on the pronotum and elytra, the outline of elytra in dorsal view is more robust, and the shape of the penis is characteristic.

**Figures 108–121.**
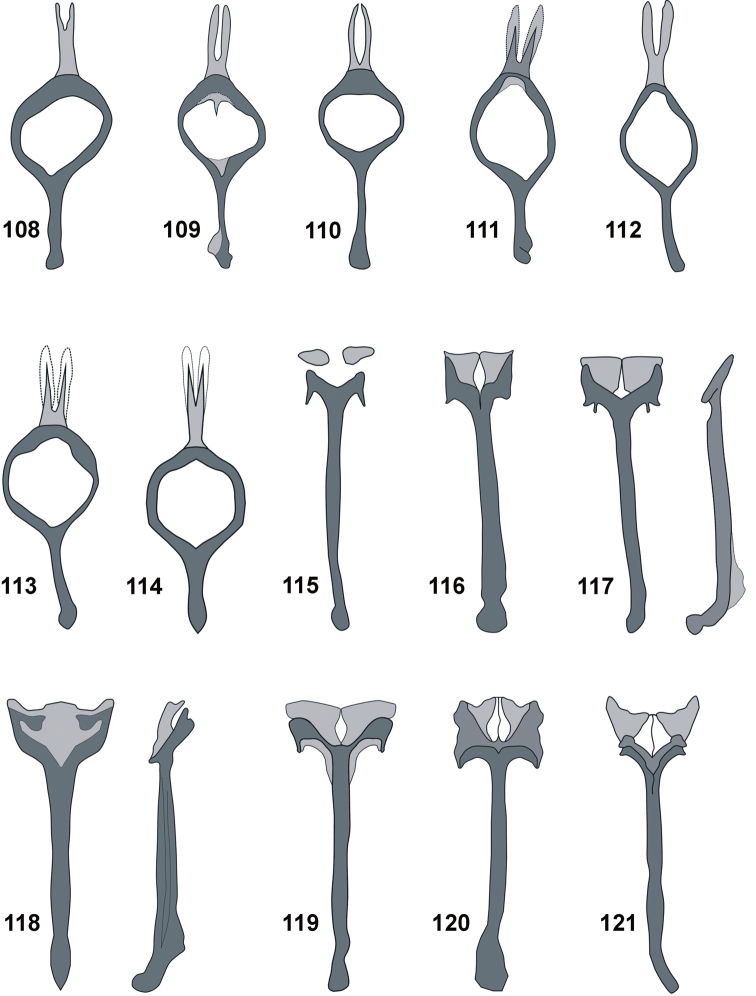
Male. Tegmen: **108***C.farinosus* Perr. **109***C.fundatus* sp. n. **110***C.gibbus* sp. n. **111***C.malleus* sp. n. **112***C.rutai* sp. n. **113***C.torosus* sp. n. **114***C.turbidus* sp. n. Spiculum gastrale: **115***C.farinosus* Perr. **116***C.fundatus* sp. n. **117***C.gibbus* sp. n. **118***C.malleus* sp. n. **119***C.rutai* sp. n. **120***C.torosus* sp. n. **121***C.turbidus* sp. n.

### Callistomorphus szoltysi sp. n.

Taxon classificationAnimaliaColeopteraCurculionidae

http://zoobank.org/9A493952-9F21-4C2C-B786-21A31DCBCBDD

Figs 19 , 28 , 37 , 46 , 55 , 64 , 73 , 82 , 91 , 125 , 130 , 134 , 138 , 143

#### Diagnosis.

Together with *C.farinosus* Perr. and *C.torosus* sp. n. this new species is one of the largest members of the genus. Easy to distinguish by several features: body colour generally whitish; apical margin of pronotum distinctly rounded in lateral view, slightly concave in dorsal view; antennae slender with long, protruding setae; medial tubercles on elytra relatively small; elytra strongly convex in lateral view. Male abdominal sternite VIII with short apodeme, apical lobe enlarged.

#### Description.

Body length (lb) – 12.30 mm.

*Body colour and vestiture* (Fig. 19). Generally whitish, with elytra entirely speckled with small, light-brown irregular spots. Pronotum with elongate light-brown spot near medial tubercles; sides darker.

*Head* (Figs 64, 73, 82). Slightly wider than long (hw/hl ♀: 1.08). Frons narrower than double width of eye. Eyes convex, as long as half length of head (eyl/hl ♀: 0.50), not protruding above margin of head in lateral view. Rostrum longer than pronotum (rl/pl ♀: 1.20); approximately 3.35 × as long as maximum width at apex (rl/arw), distinctly, regularly curved; longitudinal carina on rostrum distinct, shining on entire length, evanescent before apex of rostrum. Scape shorter than rostrum (scl/rl ♀: 0.80). Funicle slender, all antennomeres longer than wide, with elongate, straight, distinctly protruding setae; first funicle segment approximately 1.20 × as long as 2^nd^; 3^rd^ 0.7 × as long as 2^nd^; antennomeres from 4^th^ to 7^th^ with similar length, 7^th^ distinctly wider than 6^th^. Club 2.75 × as long as wide, longer than last four funicle segments combined.

**Figures 122–134.**
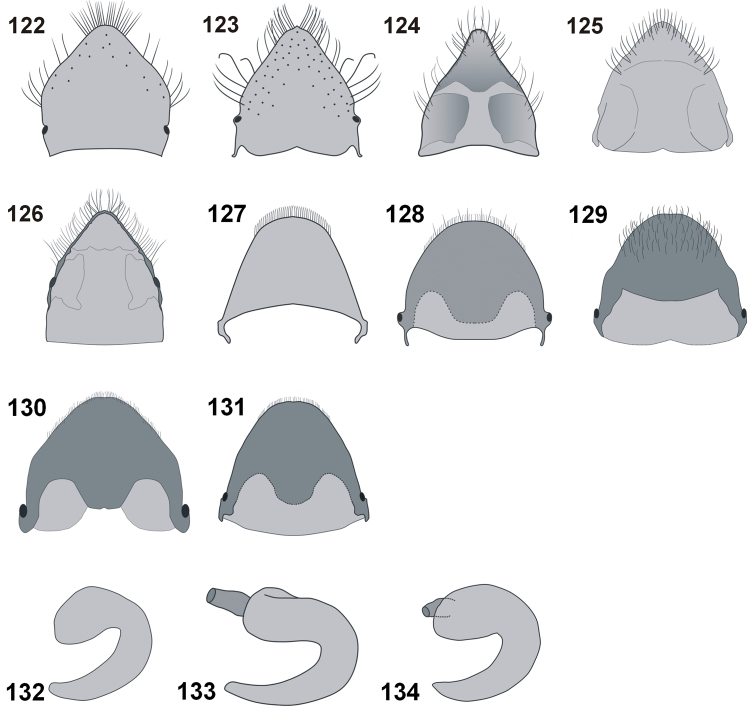
Female. Abdominal tergite VIII: **122***C.farinosus* Perr. **123***C.malleus* sp. n. **124***C.minimus* sp. n. **125***C.szoltysi* sp. n. **126***C.torosus* sp. n. Pygidium: **127***C.farinosus* Perr. **128***C.malleus* sp. n. **129***C.minimus* sp. n. **130***C.szoltysi* sp. n. **131***C.torosus* sp. n. Spermatheca: **132***C.farinosus* Perr. **133***C.malleus* sp. n. **134***C.szoltysi* sp. n.

*Pronotum* (Figs 46, 55). Wider than long (bpw/pl ♀: 1.20). Apical margin in dorsal view slightly concave with distinct, numerous tubercles, apical margin distinctly rounded in lateral view; base ca 1.43 × as wide as apical margin (bpw/apw). Medial tubercles on pronotal disc distinctly elevated, portions of pronotal disc without medial tubercles distinctly concave (easily visible when viewed laterally). Width of medial constriction in relation to apical and basal margin in female: mpw/apw = 0.67, mpw/bpw = 0.47.

*Elytra* (Figs 28, 37). Slightly more than 1.50 × as long as its width (el/bew ♀: 1.55); subparallel from base to apical part; posterior calli well developed, protruding beyond outline of elytral in dorsal view; in lateral view strongly convex. Odd intervals with distinct, pointed tubercles furnished with single, hooked, elongate scale; these tubercles are easily visible in dorsal and lateral view. Striae formed by oval punctures, each with single, whitish scale inside. Medial tubercles relatively small, short and weakly elevated. Scutellum slightly longer than wide; surrounded by narrow, asetose area, this in turn surrounded by elongate concentrically oriented scales.

*Abdomen* (Fig. 91, 130). Subquadrate, al/aw 0.98. Last ventrite 2.27 × wider than long (lvw/lvl); medial area with wide, shallow cavity, above cavity small, slightly elevated, single tubercle. Apical margin with distinct, easily visible sharp edge; slightly concave apically. Pygidium wider than long, as in Fig. 130.

*Female terminalia* (Figs 125, 134, 138, 143). Abdominal sternite VIII with enlarged apical lobe, as long as half-length of apodeme; medially with wide, distinctly sclerotised area; sides from half of length to apex with distinct punctures, each bearing short, apically hooked setae. Abdominal tergite VIII subtriangular, apically with numerous, elongate setae. Spermatheca as in Fig. 137. Ovipositor with stout gonocoxite, stylus short.

*Male* – unknown

*Measurements*. ♀: al 4.50, apw 2.10, arw 0.90, aw 4.60, bew 5.50, bpw 3.00, el 8.50, eyl 0.60, frw 0.65, hl 1.50, hw 1.40, lb 12.30, lvl 1.10, lvw 2.50, mpw 1.40, pl 2.50, rl 3.00, scl 2.40.

#### Type material.

Holotype, ♀ (here designated) – New Caledonia (S); 22°05.9'S, 166°40.7'E; Rivière Bleue Parc Kaori géant, 180 m; humid forest, 22.12.2006, rainforest; leg. R. Dobosz & M. Wanat. Additional museums (USMB) label – 5958/848. (MNHN).

#### Etymology.

With great pleasure I dedicate this species to Henryk Szołtys (Brynek, Poland), excellent coleopterologist, field researcher and my first entomology teacher.

#### Remarks.

This large member of the genus is easy to distinguish from other similarly-sized species (*C.farinosus* Perr. and *C.torosus* sp. n.) by the whitish colour of the dorsal vestiture, funicle antennomeres with protruding, elongate setae, the robust pronotum and distinctly smaller medial tubercles on elytra.

### Callistomorphus torosus sp. n.

Taxon classificationAnimaliaColeopteraCurculionidae

http://zoobank.org/A67A412B-AD00-48A1-8F00-ECEA8B4BA6AD

Figs 20 , 29 , 38 , 47 , 56 , 65 , 74 , 83 , 92 , 99 , 106 , 113 , 120 , 126 , 131 , 139 , 144

#### Diagnosis.

Together with *C.farinosus* Perr. and *C.szoltysi* sp. n. it is one of the largest members of the genus. Body uniformly dark brown. Eyes weakly convex. Elytra in lateral view weakly convex; medial tubercles large; in dorsal view sides of elytra with distinctly protruding small tubercles. Apical part of penis in lateral view strongly upturned, narrowed, apically pointed. Ovipositor gonocoxite and stylus of similar length, set diagonally to each other.

**Figures 135–144.**
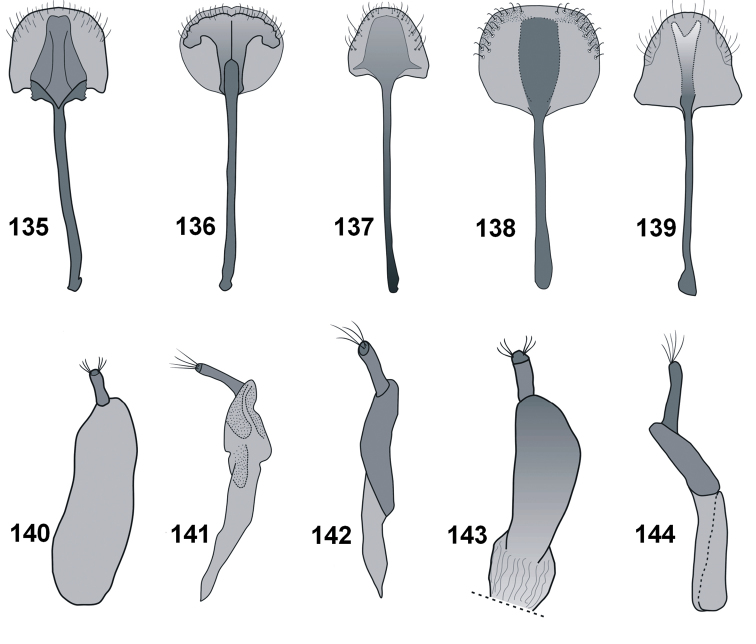
Female. Abdominal sternite VIII: **135***C.farinosus* Perr. **136***C.malleus* sp. n. **137***C.minimus* sp. n. **138***C.szoltysi* sp. n. **139***C.torosus* sp. n. Ovipositor: **140***C.farinosus* Perr. **141***C.malleus* sp. n. **142***C.minimus* sp. n. **143***C.szoltysi* sp. n. **144***C.torosus* sp. n.

#### Description.

Body length – 11.30–12.00 mm.

*Body colour and vestiture* (Fig. 20). Generally dark brown. Indistinct, darker, subtriangular spot between intervals 1–3 situated anteriorly to medial tubercles. White spot on last two intervals present, extended from approximately one-third to two-thirds of length. Apical part of mesepimeron whitish, in contrast to generally dark brown colour of body. Ventral part (metaventrite and ventrites) densely covered by light, variable (from whitish to yellowish) scales.

*Head* (Figs 65, 74, 83). Subquadrate in female, slightly shorter than wide in male (hw/hl ♂: 0.92; ♀: 1.00). Eyes flattened, slightly in male, more distinctly in female; shorter than half length of head (eyl/hl ♂: 0.38; ♀: 0.46); not protruding above margin of head in lateral view. Frons as wide as double width of eyes or slightly wider. Rostrum slightly longer than pronotum (rl/pl ♂: 1.12; ♀: 1.07); from 3.33 (female) to 3.50 (male) × as long as maximum width at apex (rl/arw), distinctly, regularly curved; longitudinal carina on rostrum distinct, medially covered with scales, surface between eyes and on apical part of rostrum shining. Scape shorter than rostrum (scl/rl ♂: 0.82; ♀: 0.83). First funicle segment 1.60 × as long as 2^nd^; 3^rd^ 0.65 × as long as 2^nd^; 4^th^ slightly longer than wide; from 5^th^ to 7^th^ as long as wide. Club slender, 2.50 × as long as wide; as long as last four funicle segment combined. Setae on antennomeres distinct, elongate, moderately protruding.

*Pronotum* (Figs 47, 56). Subquadrate in male, slightly longer than wide in female (bpw/pl ♂: 1.04; ♀: 1.11). Apical margin straight with distinct tubercles; base from 1.30 × (♂) to 1.35 × (♀) as wide as apical margin (bpw/apw). Medial tubercles distinct, strongly protruding, apically obtuse. Width of medial constriction in relation to apical and basal margin: mpw/apw = 0.61 (♀), 0.65 (♂); mpw/bpw = 0.45 (♀), 0.50 (♂).

*Elytra* (Figs 29, 38). Slightly more than 1.50 × as long as its width (el/bew ♂: 1.51; ♀: 1.55). Subparallel from base to apical part; 7^th^ interval with strongly protruding tubercle before apex of elytra – posterior calli well developed protruding beyond outline of elytral in dorsal view; in lateral view elytra weakly convex. Odd intervals with distinct tubercles, that are pointed on basal half of elytral disc, and more obtuse on apical part. Striae easily visible, formed by distinct oval punctures. Medial tubercles large; slightly longer than one-quarter length of elytra; in lateral view, more or less one-third of elytral height medially. Scutellum short, subquadrate.

*Abdomen* (Figs 92, 106, 131). Subquadrate in male, slightly shorter than wide in female (al/aw ♂: 1.00; ♀: 0.93). Last ventrite much wider than long (lvw/lvl ♂: 2.56; ♀: 2.78); both sexes with sharp apical margin; in male apical area distinctly concave, in female apical cavity deeper. Pygidium of male as in Fig. 106, female as in Fig. 131.

*Male terminalia* (Figs 99, 113, 120). Penis body longer than apodemes; from base to fourth-fifth of length slightly dilated, remainder of penis distinctly narrowed to rounded apex, its basal part sclerotised; in lateral view strongly curved, apically distinctly upward. Internal sac without any distinct structure or sclerites. Parameroid lobes of tegmen divided almost from base. Spiculum gastrale anchor-shaped; hemisternites well sclerotised, fused with base of spiculum.

*Female terminalia* (Figs 126, 139, 144). Abdominal sternite VIII forked apically, apical lobe enlarged, sclerotised on sides, with erect setae. Abdominal tergite VIII elongate; apex widely rounded. Ovipositor – gonocoxite and stylus of similar length, set diagonally to each other. Spermatheca lost in dissection.

*Measurements*. ♂: al 4.10, apw 2.00, arw 0.80, aw 4.10, bew 4.90, bpw 2.60, el 7.20, eyl 0.50, frw 0.60, hl 1.30, hw 1.20, lb 11.00, lvl 0.90, lvw 2.30, mpw 1.30, pl 2.50, rl 2.90, scl 2.30.

♀: al 4.30, apw 2.30, arw 0.85, aw 4.60, bew 5.30, bpw 3.10, el 8.00, eyl 0.60, frw 0.60, hl 1.30, hw 1.30, lb 12.00, lvl 0.90, lvw 2.50, mpw 1.40, pl 2.80, rl 3.00, scl 2.50.

#### Type material.

Holotype, ♂ (here designated) – New Caledonia (N); 20°23.9'S, 164°32.0'E; Mandjélla (subsummit), 11.01.2007, 700–750 m, night beating; leg. M. Wanat & R. Dobosz (MNHN).

Paratype, ♀ – New Caledonia (N); 20°23.9'S, 164°31.9'E; Mandjélla (summit), 10.01.2007, 750–780 m, beating, montane rainforest; leg. M. Wanat & R. Dobosz (MNHW).

#### Etymology.

This epithet is derived from the Latin adjective “*torosus*” (muscular) and refers to “muscular” shape and size.

### Callistomorphus turbidus sp. n.

Taxon classificationAnimaliaColeopteraCurculionidae

http://zoobank.org/12FC9A5D-67E0-4F51-A97C-DA725829B1FB

Figs 5 , 11 , 21 , 30 , 39 , 48 , 57 , 66 , 75 , 84 , 93 , 100 , 107 , 114 , 121

#### Diagnosis.

Easy to distinguish by combination of several features: apical and basal margin of pronotum slightly concave, apical margin in lateral view distinctly protruding towards head; rostrum relatively short and stout, less than 3 × as long as maximum width apically; strongly curved; penis strongly upwards before two-thirds of length; pygidium apically with distinct depression in ventral view; lateral margin of pygidium in ventral view irregular.

#### Description.

Body length (lb) – 8.50 mm.

*Body colour and vestiture* (Fig. 21). Generally dark brown. Indistinct, paler, stripe from scutellum to base of medial tubercles on third intervals; oblique paler stripe behind medial tubercles reaching paler spot on side of elytra. Pronotum with two, indistinct, lighter, narrow, longitudinal stripes of scales. Head and rostrum covered with paler scales. Tibiae orange, distinctly paler than dark brown femora. Ventrites dark brown with bundle of paler scales on 3^rd^ and 4^th^ ventrites.

*Head* (Figs 5, 66, 75, 84). Slightly longer than wide (hw/hl ♂: 0.92). Eyes convex with maximum width before middle; shorter than half length of head (eyl/hl ♂: 0.42); not protruding above margin of head in lateral view. Frons slightly wider than double width of eyes. Rostrum slightly longer then pronotum (rl/pl ♂: 1.11); strongly curved and stout (rl/arw ♂: 2.86); longitudinal carina on rostrum very distinct, sharp and polished from base to antennal insertion. Scape shorter than rostrum (scl/rl ♂: 0.75). First funicle segment short, only 1.2 × as long as 2^nd^; 3^rd^ 0.6 × as 2^nd^; from 3^rd^ to 7^th^ with similar length; club as long as last four funicle segment combined; setae on antennomeres distinct, elongate, moderately protruding.

*Pronotum* (Figs 48, 57). Slightly wider than long (bpw/pl ♂: 1.17). Base 1.24 × as wide as apical margin (bpw/apw); apical margin concave with weakly developed tubercles, only on apical angles tubercles distinct and protruding; in lateral view apical margin protruding towards head; basal margin slightly, but visibly, concave medially; basal angles, in dorsal view, lying clearly below middle part of basal margin. Medial tubercles weakly developed, obtuse; in lateral view slightly protruding above margin of pronotum. Width of medial constriction in relation to apical and basal margin in male: mpw/apw = 0.59; mpw/bpw = 0. 48.

*Elytra* (Figs 30, 39). Relatively short (el/bew ♂: 1.47); slightly narrowed behind humeral angles; posterior calli developed, protruding beyond outline of elytra in dorsal view. Surface with very rough sculpture; striae composed of subcircular, shallow punctures; surface bordering striae and intervals indistinct, especially on basal half in front of medial tubercles. Apically striae evanescent, only as shallow punctures. Medial tubercles distinct, lower than width at base. Scutellum slightly longer than wide.

*Abdomen* (Figs 93, 107). Slightly longer than wide (al/aw ♂: 1.06). Last ventrite 2.13 × wider than long (lvw/lvl); apical margin sharp; medially on apical portion wide, shallow cavity. Pygidium with a specific shape; apically, in ventral view, with deep cavity.

*Male terminalia* (Figs 100, 107, 114 121). Penis body distinctly longer than apodemes; base fully sclerotised; from base slightly dilated, maximum width before midlength; before two-thirds of length distinctly narrowed, then subparallel to widely rounded apex; distinctly curved in lateral view, upward before two-thirds of length. Internal sac without any visible structures or sclerites. Parameroid lobes and tegminal apodeme with similar length; divided beyond middle of length. Spiculum gastrale Y-shaped; hemisternites fused with base of spiculum. Hemisternites of sternite VIII elongate, clavate.

*Female* – unknown

*Measurements*. ♂: al 3.40, apw 1.70, arw 0.70, aw 3.20, bew 3.80, bpw 2.10, el 5.60, eyl 0.50, frw 0.55, hl 1.20, hw 1.10, lb 8.50, lvl 0.80, lvw 1.70, mpw 1.00, pl 1.80, rl 2.00, scl 1.50.

#### Type material.

Holotype, ♂ (here designated) – New Caledonia (N); 20°24'00.3"S, 164°31'40.4"E; Mt. Mandjélla 700–780 m; montane rainforest; 20.11.2008, leg. M. Wanat. (MNHN).

#### Etymology.

This epithet is derived from the Latin adjective “*turbidus*” (confused, impatient) and refers to my feelings after I wasted too much time trying to create any suitable name for this creature.

#### Remarks.

By the short, distinctly curved rostrum, small size and very characteristic male terminalia (unique form of pygidium, strongly upwardly-directed penis body in lateral view), this species is easy to distinguish within the genus. A female is unknown but may be easily to distinguished based on the description presented above.

**Figure 145.**
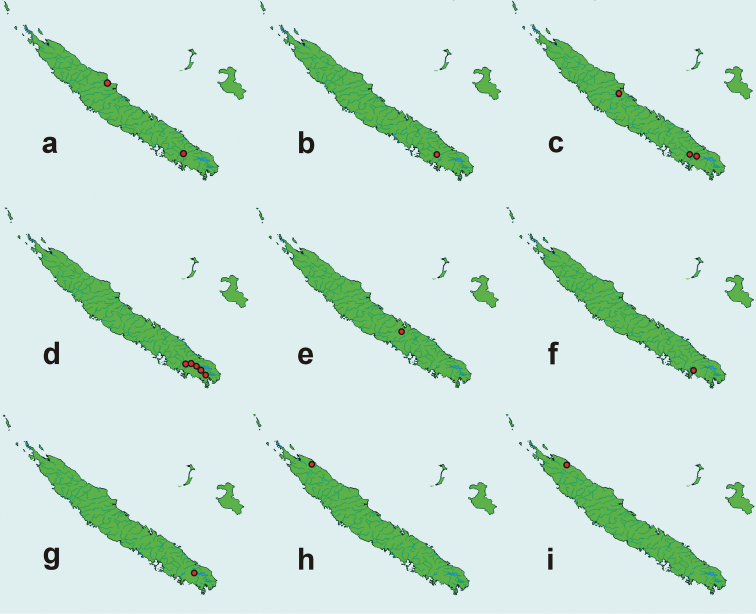
Distribution maps of New Caledonian species of *Callistomorphus*: **a***C.farinosus* Perr. **b***C.fundatus* sp. n. **c***C.gibbus* sp. n. **d***C.malleus* sp. n. **e***C.minimus* sp. n. **f***C.rutai* sp. n. **g***C.szoltysi* sp. n. **h***C.torosus* sp. n. **i***C.turbidus* sp. n.

### Key to species of the genus *Callistomorphus*

**Table:** 

1	Elytra and pronotum glabrous, without prominent tubercles (Figs 26, 35); eyes strongly convex, distinctly protruding above margin of head in lateral view (Fig. 62); length of body less than 7.50 mm	***C.minimus* sp. n.**
–	Elytra and pronotum strongly scabrous with distinct medial tubercles and numerous, small tubercles on entire elytra (e.g. Figs 25, 33); eyes more or less convex but not protruding above margin of head in lateral view (e.g. Fig. 58); body length greater than 7.50 mm	**2**
2	Apical margin of pronotum strongly concave in dorsal view, protruding towards head in lateral view (e.g. Figs 42, 51)	**3**
–	Apical margin of pronotum straight or slightly concave in dorsal view, not protruding towards head in lateral view (e.g. Figs 43, 52)	**6**
3	Body length greater than 10 mm; medial tubercles on elytra relatively short, subequal to one-fifth of elytral length (e.g. Fig. 37)	**4**
–	Body length less than 10 mm; medial tubercles relatively elongate, subequal to one-third of elytral length (e.g. Fig. 33)	**5**
4	Medial tubercles on pronotum weakly protruding, obtuse (Fig. 49); body generally dark brown with distinct, large, whitish spot on middle of elytra (Fig. 13)	*** C. farinosus ***
–	Medial tubercles on pronotum strongly protruding, rounded (Fig. 55); body generally whitish without any distinct spot on elytra (Fig. 19)	***C.szoltysi* sp. n.**
5	Base of pronotum medially rounded (Fig. 42), medial tubercles on pronotum strongly protruding, rounded (Fig. 51); rostrum elongate, more than 3 × as long as wide (rl/arw = 3.00–3.30)	***C.gibbus* sp. n.**
–	Base of pronotum slightly concave (Fig. 48), medial tubercles on pronotum weakly protruding, obtuse (Fig. 57); rostrum short, less than 3 × as long as wide (rl/arw = 2.85)	***C.turbidus* sp. n.**
6	Rostrum weakly curved, almost straight, slightly narrowed to apex, 4.00 × as long as maximum width or longer (e.g. Fig. 61)	**7**
–	Rostrum regularly curved, indistinctly narrowed to apex, 3.30–3.60 × as long as maximum width (e.g. Fig. 59)	**8**
7	Medial tubercles on elytra short, less than 2 × width of intervals in the middle of elytra; numerous, small tubercles on entire elytra mostly obtuse (Fig. 34); penis body apically expanded into small tubercles in lateral view (Fig. 97)	***C.malleus* sp. n.**
–	Medial tubercles on elytra tall, greater than 2 × width of intervals in the middle of elytra; numerous, small tubercles on entire elytra mostly sharp, pointed (Fig. 36); penis body apically rounded in lateral view (Fig. 98)	***C.rutai* sp. n.**
8	Elytra elongate, 1.64 × as long as wide (Fig. 23); base of pronotum slightly concave; last ventrite of male subtriangular, 1.86 × wider than long (Fig. 86)	***C.fundatus* sp. n.**
–	Elytra shorter, 1.55 × as long as wide (Fig. 29); base of pronotum straight; last ventrite in both sexes shorter, 2.20–2.30 × wider than long (Fig. 92)	***C.torosus* sp. n.**

For a clear presentation of measurements, important for distinguishing particular species, all indices are presented in Table 1. All available specimens have been measured.

## Taxonomic position of the genus *Callistomorphus* Perroud

As was mentioned in the introduction, the genus *Callistomorphus* was forgotten or ignored for decades in most of the previously published research on Eugnomini.

Currently, the tribe seems to be not monophyletic, without any clear synapomorphies uniting all the genera. Voss (1937) and Marshall (1937) indicated that the essential feature is the elongation and flexibility of the maxillary palpi, but at the same time they emphasised the weakness of this feature for defining Eugnomini, as it does not occur in the genus *Pactola*. Short maxillary palpi are also present in a close relative of *Pactola* – *Pactolotypus* Broun, 1909, as well as in some species of *Eugnomus* and *Rasilinus* Mazur, 2016 (Mazur 2016 and Mazur – unpublished data). Marshall (1937) distinguished several other features that he suggested as characteristic for Eugnomini, but there are numerous exceptions if we assume the genera currently included in the tribe (Alonso-Zarazaga and Lyal 1999). However, most of these features are represented in *Callistomorphus*.

Cawthra (1966) redefined Eugnominae (sensu Voss 1937) and established a systematic positioning of the tribe, which was subsequently adopted by Alonso-Zarazaga and Lyal (1999) with minor changes. Additionally, Cawthra (1966) included the five genera of Meriphinae Marshall, 1937 in Eugnominae, which are currently treated as a subtribe of Eugnomini (Alonso-Zarazaga and Lyal 1999). The distinguishing characteristics of Eugnomini sensu Cawthra (1966) (without Meriphina), with their relationship to *Callistomorphus* and the exceptions within the tribe, are presented below.

1. Maxilla with elongate second segment of the palpus – present in *Callistomorphus*; maxilla not elongate in some *Eugnomus*, *Pactola*, *Pactolotypus*, *Rasilinus*, *Udeus* Champion, 1902.

2. Head elongate behind the eyes with the temples as long as, or longer than, the eyes – present in *Callistomorphus*; head not elongate in: *Koghicola* Mazur, 2014, *Omoides* Boheman, 1859, some *Pactola*, *Pactolotypus*, *Udeus*.

3. Antennal scrobes oblique, turning rapidly downwards and continued on the lower side of rostrum – present in *Callistomorphus*; within Eugnomini different (not continued) only in *Goneumus* Marshall, 1937.

4. Funicle with seven antennomeres – present in *Callistomorphus*; six-segmented in *Nyxetes* Pascoe, 1870, *Oreocalus* May, 1993, *Pactolotypus*.

5. Hind wings well developed – present in *Callistomorphus* and all other genera except flightless *Pactolotypus*, some *Eugnomus* and *Stephanorhynchus*.

6. Elytra with large tubercles or conspicuous cones – very characteristic for many genera, including *Callistomorphus* also, but absent in some others, including: *Ancyttalia* Zimmerman, 1994, *Eugnomus*, *Goneumus*, *Hoplocneme*, *Koghicola*, *Omoides*, *Oreocalus*, some *Pactola*, *Pactolotypus*, *Rhopalomerus*, *Tysius* Pascoe, 1875, *Udeus*.

7. Front coxae contiguous – present in *Callistomorphus*, but coxae separate (sometimes slightly) in *Gonoropterus* Broun, *Omoides*, *Pactolotypus* and *Udeus*.

8. At least posterior femora distinctly extended, strongly toothed – characteristic also for *Callistomorphus*, weakly extended with small tooth in *Ancistropterus* White, 1846, *Eugnomus* and *Goneumus*.

9. Hind tibiae strongly, regularly curved or distinctly sinuate – present in *Callistomorphus*, hind tibiae straight in many genera, including: *Ancistropterus*, *Ancyttalia*, *Eugnomus*, *Goneumus*, *Hoplocneme*, *Icmalius* Broun, 1893, *Nyxetes*, *Pactolotypus*, *Rhopalomerus*, *Scolopterus*, *Tysius*, *Udeus*.

10. Apex of fore tibiae not mucronate in male – lack of mucro in *Callistomorphus*, tibiae are mucronate in *Ancistropterus*, *Omoides*, some *Rhopalomerus* and *Udeus*.

*Callistomorphus* is a genus that seems to be closely related to *Stephanorhynchus*, as was previously suggested by Voss (1936). In both genera, the head is distinctly constricted near the base, the claws are unarmed, and all the characteristics mentioned above are shared. Additionally, the structures of the medial tubercles on the elytra and the shapes of the legs are similar (Characteristics 8–10, see above). Both genera are easy to distinguish by the shape of the rostrum (elongate in *Callistomorphus* and short in *Stephanorhynchus*); the mandible (of a normal size and overlapping in *Stephanorhynchus*); dorsal part of rostrum (better developed and pronounced ridge in *Stephanorhynchus*); pronotum (subapical constriction only slightly marked in *Stephanorhynchus*); anterior surface of pronotum (not flattened in *Stephanorhynchus*).

Currently, Eugnomini needs a detailed revision and a comprehensive diagnosis. Since the last study of Cawthra (1966), some genera were included in Eugnomini but these actions were in some cases questioned by other authors (e.g. the genus *Apionodes* Marshall, 1948 was transferred from Anthonomini to Eugnomini by Kojima and Morimoto (1993) and was then shifted back to Anthonomini by Alonso-Zarazaga and Lyal (1999), currently it is a synonym of *Pseudopoophagus* Voss, 1935 in Eugnomini (Caldara 2013); *Oropterus* White, 1846 was incorrectly transferred to Eugnomini by Alonso-Zarazaga and Lyal (2002); and *Acanthopterus* was transferred from Eugnomini to Aterpini, without the appropriate argumentation, by Kuschel (2014)). The lack of a clear diagnosis and the unrecognised taxonomic status of some genera makes further research difficult, especially in the area of New Caledonia, where many undescribed taxa strongly resemble Eugnomini sensu Cawthra (1966). Revisions of all the genera currently placed within the tribe will allow the connections between the three main areas inhabited by the Eugnomini – Australia, New Zealand and New Caledonia – and their relationships with the single, specific genera (*Omoides*, *Udeus*) occurring only in the New World to be examined.

## Supplementary Material

XML Treatment for Callistomorphus

XML Treatment for Callistomorphus farinosus

XML Treatment for Callistomorphus fundatus

XML Treatment for Callistomorphus gibbus

XML Treatment for Callistomorphus malleus

XML Treatment for Callistomorphus minimus

XML Treatment for Callistomorphus rutai

XML Treatment for Callistomorphus szoltysi

XML Treatment for Callistomorphus torosus

XML Treatment for Callistomorphus turbidus
